# Dihydroisatropolone C from *Streptomyces* and Its Implication in Tropolone-Ring Construction for Isatropolone Biosynthesis

**DOI:** 10.3390/molecules27092882

**Published:** 2022-04-30

**Authors:** Jiachang Liu, Xiaoyan Liu, Jie Fu, Bingya Jiang, Shufen Li, Linzhuan Wu

**Affiliations:** NHC Key Laboratory of Biotechnology of Antibiotics, CAMS Key Laboratory of Synthetic Biology for Drug Innovation, Institute of Medicinal Biotechnology, Chinese Academy of Medical Sciences & Peking Union Medical College, Tiantan Xili, Beijing 100050, China; ljchang_1019@163.com (J.L.); lxyzjk445@163.com (X.L.); cfujie@126.com (J.F.); jiangbingya@163.com (B.J.)

**Keywords:** *Streptomyces*, 7,12-dihydroisatropolone C, spontaneous oxidation

## Abstract

Isatropolones/isarubrolones are actinomycete secondary metabolites featuring a tropolone-ring in their structures. From the isatropolone/isarubrolone producer *Streptomyces* sp. CPCC 204095, 7,12-dihydroisatropolone C (H_2_ITC) is discovered and identified as a mixture of two interchangeable diastereomers differing in the C-6 configuration. As a major metabolite in the mycelial growth period of *Streptomyces* sp. CPCC 204095, H_2_ITC can be oxidized spontaneously to isatropolone C (ITC), suggesting H_2_ITC is the physiological precursor of ITC. Characterization of H_2_ITC makes us propose dihydrotropolone-ring construction in the biosynthesis of isatropolones.

## 1. Introduction

Isatropolones/isarubrolones are a complex of actinomycete secondary metabolites featuring a tropolone-ring in their structures, with isarubrolones being the non-enzymatic conjugation products of isatropolones with amines or amino acids [1]. Rubrolones and rubterolones are also actinomycete secondary metabolites featuring a tropolone-ring in their structures [2,3,4]. These metabolites share very similar structures and biosynthetic pathways [1,5,6]. In particular, the tropolone-rings in these metabolites are all constructed by enzyme-catalyzed oxidative rearrangement of poly-β-ketoacyl intermediates from type-II polyketide synthase (PKS) pathways [5]. However, tropolone-ring construction in the biosynthesis of these secondary metabolites has not been chemically confirmed, possibly because intermediates appearing in the tropolone-ring construction process are rather unstable.

We are interested in novel secondary metabolites, together with their biosynthesis, from actinomycetes [7,8,9]. Previously, we identified an isatropolone/isarubrolone producer *Streptomyces* sp. CPCC 204095 and discovered autophagic activity for the isatropolones/isarubrolones characterized from the strain. In a recent study of isatropolones/isarubrolones and their production by *Streptomyces* sp. CPCC 204095, we identified 7,12-dihydroisatropolone C (H_2_ITC) as a novel component of isatropolones, and determined it as the precursor of isatropolone C (ITC). In particular, characterization of H_2_ITC led us to present a revised tropolone-ring construction process in isatropolone (and rubrolone) biosynthesis that had been proposed before [1,5].

## 2. Results and Discussion

### 2.1. Discovery of 7,12-Dihydroisatropolone C (H_2_ITC) from Streptomyces *sp.* CPCC 204095

**Figure 1:**
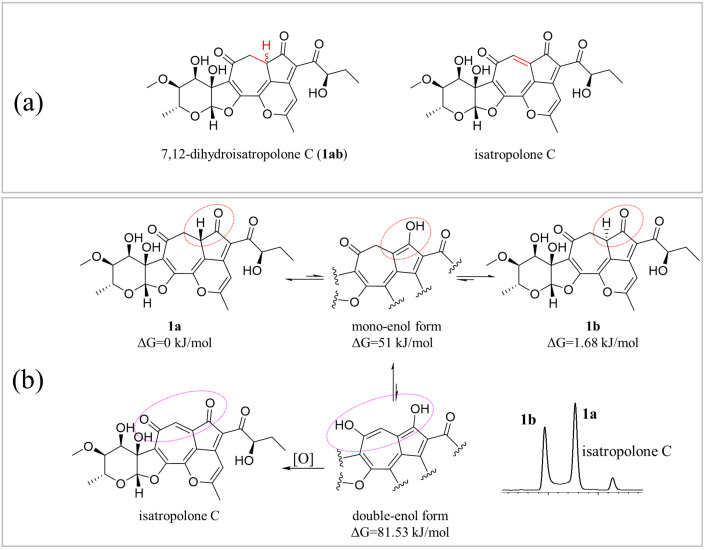
The structure of 7,12-dihydroisatropolone C (H_2_ITC, **1ab**) and its oxidation to isatropolone C (ITC). (**a**) 7,12-dihydroisatropolone C and isatropolone C. (**b**) Keto-enol tautomerization of **1a** and **1b** in H_2_ITC, and their spontaneous oxidation to isatropolone C. The HPLC trace showed the pair peaks of H_2_ITC (**1ab**) and a small isatropolone C peak.

Previously, we identified the isatropolone/isarubrolone producer *Streptomyces* sp. CPCC 204095, with isatropolone C (Figure 1a) as a major component [10]. In the exploration of more metabolites with a tropolone-ring from the producer, we observed one HPLC pair peaks with identical but novel UV–visible absorption from an acidified ethyl acetate (EtOAc-5% acetic acid) extract of fermentation culture of the producer (Figures S1, S4 and S5). In addition, the acidified EtOAc extract produced a lower isatropolone C (ITC) peak than the EtOAc extract that gave a dominant ITC peak (Figure S2), suggesting a relationship between the pair peaks and the ITC peak. The pair peaks aroused our interest in their identities.

### 2.2. Structural Elucidation of 7,12-Dihydroisatropolone C (H_2_ITC)

The pair peaks exhibited the same molecular mass (*m*/*z* at 475 for [M+H]^+^) and MS^2^ fragmentation pattern in LC-MS analysis (Figure S6), indicating two isomeric molecules in the pair peaks. When the pair peaks were separated by HPLC, each peak would change back instantly to the former pair peaks, and the latter peak was always higher than the former peak. Therefore, compounds in the pair peaks were purified as a whole (mixture of epimers) for structure elucidation (Figure S3).

A procedure of silica gel column chromatography, ODS column chromatography and reverse phase HPLC was used to purify the compound (**1ab**) in the pair peaks. Specifically, elutes from column chromatography and HPLC were stored at low temperature (0 or −20 °C), as **1ab** was able to change slowly to ITC at room temperature. The purified **1ab** sample was kept at −20 °C, and its NMR assay was conducted at −4 °C.

Compound **1ab** was obtained as light-yellow amorphous powder. HRESIMS established its molecular formula C_24_H_26_O_10_, two hydrogen atoms more than ITC (Figure S7). Its NMR spectra indicated a major set of signals for **1ab**, together with an expected minor set of signals for ITC (Figure S8). Luckily, these minor signals did not cause much difficulty in recognizing the major signals. A close examination of major signals revealed that they were all paired ones, indicating that **1ab** was a mixture of two diastereomers.

**Figure 2:**
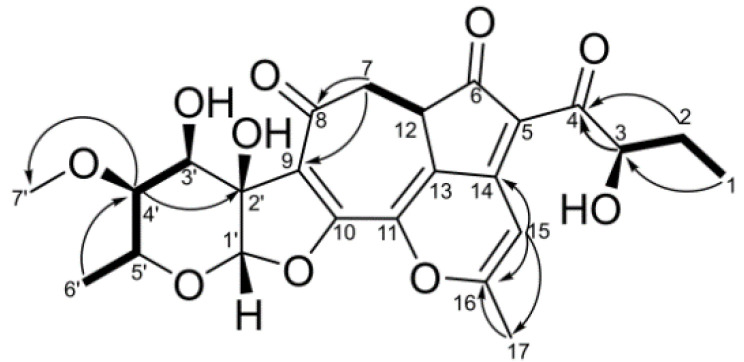
Key COSY and HMBC correlations for 7,12-dihydroisatropolone C (H_2_ITC, **1ab**).

The ^1^H and ^13^C NMR spectra of **1ab** were very similar to ITC, except that a pair methine signals [*δ*_C_ (40.48; 40.37), *δ*_H_ (2.91, d; 2.74, 2H, d)] and a pair methene signals [*δ*_C_ (42.33; 42.24), *δ*_H_ (3.65;3.56, 2H, dd)] replaced the two *sp*^2^ carbon signals [*δ*_C_ (134), *δ*_C_ (135), *δ*_H_ (7.12, s), *δ*_H_ (7.77, s)] in ITC. The structure of **1ab** was established as 7,12-dihydrogenated ITC (Figure 1a) based on detailed analysis of NMR spectra, especially the ^1^H-^1^H COSY correlations of H-7/H-12 and the HMBC correlations from H-7 to C-8 and C-9 (Figure 2). Additionally, compared with ITC, the C-6, C-8, C-11 and C-9 resonances of **1ab** were shielded by Δ*δ*_C_ −2.4, −1.6, −2.8 and −0.5 ppm, respectively, which further supported **1ab** as 7,12- dihydroisatropolone C (H_2_ITC). The NMR data of H_2_ITC (**1ab**) were assigned as in Table 1 (Figures S9–S21).

### 2.3. Keto-Enol Tautomerization of 7,12-Dihydroisatropolone C (H_2_ITC, ***1ab***)

Keto-enol tautomerizations have been reported for rubrolones and rubterolones [5,6]. A keto-enol tautomerization at C-6 of H_2_ITC (**1ab**) may result in a mixture of two diastereomers (**1a** and **1b**) differing in the chiral C-12 configuration (Figure 1b). The one with C-12 *S* configuration was designated as **1a**, and the other one with C-12 *R* configuration as **1b**. The ΔG calculated by Multiwfn at ωB97XD/TZVP level revealed only a small energy difference for **1a** and **1b** [11]. Population distribution was 66.29% for **1a** and 33.71% for **1b** when they reached equilibrium, approximately agreeing with peak area ratio of the pair peaks (Figure 1b). Thus, **1a** was assigned to the higher one, and **1b** to the lower one, of the pair peaks.

### 2.4. Spontaneous Oxidation of 7,12-Dihydroisatropolone C (H_2_ITC, ***1ab***) to Isatropolone C (ITC)

The mechanism for spontaneous oxidation of H_2_ITC to ITC was also proposed as in Figure 1b, in which keto-enol exchange occurred at both C-6 and C-8 to generate H_2_ITC double-enol form. Like phenols/hydroquinones [12], H_2_ITC double-enol form is sensitive to air (O_2_) oxidation, becoming ITC upon abstraction of two hydrogen atoms. The oxidation process was sped up at high temperature and pH, or slowed down at low temperature and pH. A careful examination of the ^1^H-NMR spectrum of H_2_ITC revealed two low-field signals at *δ*_H_ 11.5 and 11.4 for active hydrogen atoms that were further proved by deuterium exchange (Figure S22), thus confirming the existence of the H_2_ITC double-enol form in H_2_ITC.

**Figure 3:**
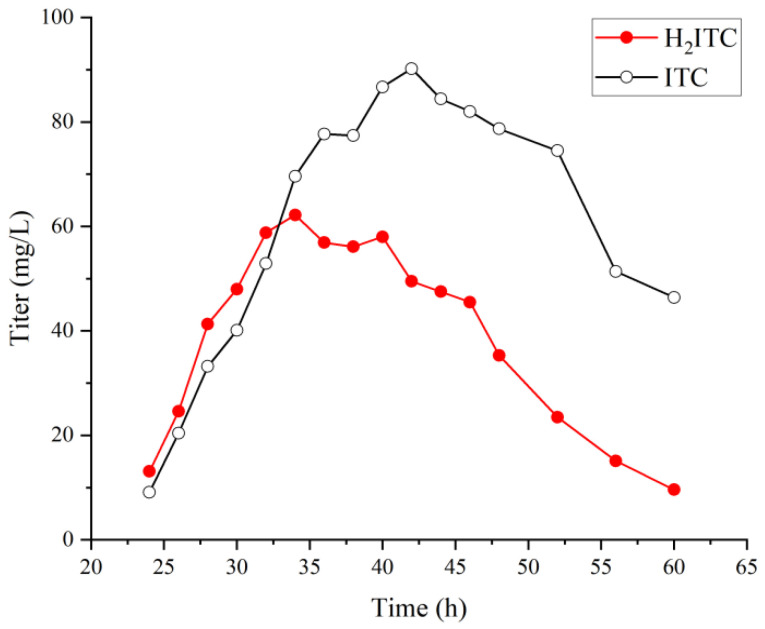
H_2_ITC and ITC titers of *Streptomyces* sp. CPCC 204095.

Spontaneous oxidation of H_2_ITC to ITC was then compared at: (a) 20 °C plus pH7.0, (b) −20 °C plus pH7.0 and (c) 20 °C plus pH8.0. A proportion of ca. 23% H_2_ITC was oxidized to ITC in 60 h at 20 °C plus pH7.0, and all H_2_ITC was oxidized to ITC in 40 min at 20 °C plus pH8.0, while H_2_ITC remained nearly unchanged for 60 h at −20 °C plus pH7.0 (Figures S25–S27). In addition, a time-course monitoring of *Streptomyces* sp. CPCC 204095 indicated a slightly higher titer of H_2_ITC than ITC in the mycelial growth period (26–32 h) of the strain, then a higher titer of ITC than H_2_ITC afterwards due to H_2_ITC oxidation to ITC and its accumulation (Figure 3; ITC titer declined after 45 h due to ITC conjugating amines and amino acids for isarubrolone production). These results indicate H_2_ITC is the physiological precursor of ITC.

Isatropolones are able to conjugate amines to form isarubrolones. H_2_ITC was explored for its conjugation with NH_3_ for 7,12-dihydroisarubrolone C formation. However, 7,12-dihydroisarubrolone C was not identified from H_2_ITC with NH_3_, while isarubrolone C was produced from the reaction. A possible reason for this may be that 7,12-dihydroisarubrolone C is much more sensitive to oxidation than H_2_ITC, so it is oxidized immediately to isarubrolone C after its formation (Figure S28).

It is very interesting that H_2_ITC can be spontaneously oxidized to ITC. A similar case has been reported for kibdelone B, a heterocyclic polyketide from *Kibdelosporangium*. Keto-enol tautomerizations of kibdelone B lead to aromatization of its ring C. Then, the aromatized intermediate, as a hydroquinone, undergoes spontaneous oxidation to convert kibdelone B with a C-C single bond in ring C to kibdelone A with a corresponding C-C double bond [13].

### 2.5. Dihydrotropolone-Ring Construction in Isatropolone Biosynthesis Implicated by H_2_ITC

As the most interesting and intriguing part in the biosynthesis of some actinomycete secondary metabolites with a tropolone-ring, Yan et al. proposed a tropolone-ring construction for rubrolones based on feeding with [^13^C]-acetate [5]. Specifically, a mono-cyclic/aromatic intermediate derived from a poly-β-ketoacyl chain underwent complex oxidative rearrangement to construct the tropolone-ring (route 1, Scheme S1), in which two oxidations at C-11 and C-12 occurred. A similar tropolone-ring construction using a bi-cyclic/aromatic intermediate was proposed for isatropolone biosynthesis by Cai et al. (route 2, Scheme S1) [1]. However, neither tropolone-ring constructions is chemically proved.

**Scheme 1:**
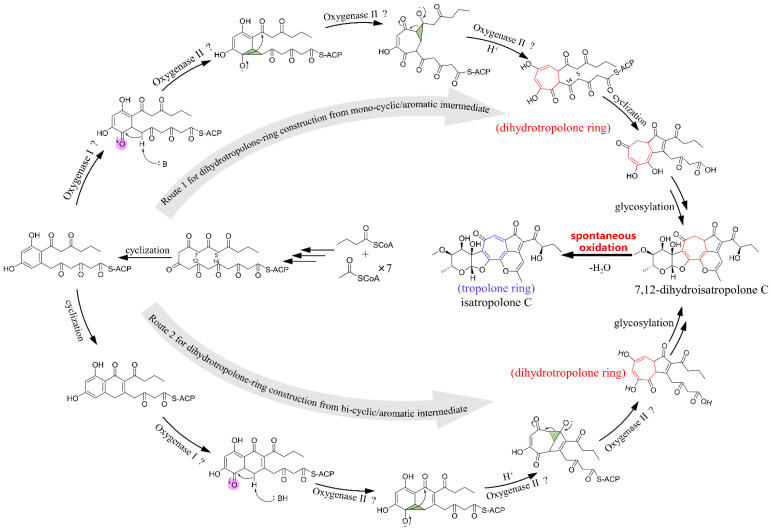
Biosynthesis of isatropolone C (ITC) focusing on oxidative rearrangement for dihydrotropolone-ring construction from mono-cyclic/aromatic intermediate (route 1) or bi-cyclic/aromatic intermediate (route 2).

The discovery of H_2_ITC from *Streptomyces* sp. CPCC 204095 and its spontaneous oxidation to ITC made us propose a dihydrotropolone-ring construction in isatropolone biosynthesis (Scheme 1). The construction involved only one oxidation at C-11 in the oxidative rearrangement. The dihydrotropolone-ring in H_2_ITC could be converted to the tropolone-ring in ITC by spontaneous oxidation.

Genetic studies have demonstrated that two oxygenase genes are essential for the oxidative rearrangement of tropolone-ring construction in rubrolone biosynthesis [14]. However, the biochemical roles of the two oxygenases are still not clear, as substrates (the mono- or bi-cyclic/aromatic intermediates with polyketoacyl chains in Scheme 1) for the two oxygenases are very unstable and difficult to prepare, which prevents in vitro characterization of the biochemical reactions they catalyze.

Cai et al. conducted a heterologous expression of *istG-R* from an isatropolone gene cluster for aglycone biosynthesis in *Streptomyces lividans*, which resulted in the identification of two aglycones (compound **10** and its reduced derivative compound **9**) [1]. Recently, Yijun Yan et al. reported multifunctional and non-stereoselective oxidoreductase RubE7/IstO for oxidation and reduction of these aglycones [15]. Our discovery of H_2_ITC seems to suggest that the primary aglycone in isatropolone biosynthesis may be a dihydrogenated **10**. Its glycosylation (and hydroxylation) in *Streptomyces* sp. CPCC 204095 leads to the production of H_2_ITC, whose spontaneous oxidation generates ITC (Scheme 1, Figure S29).

## 3. Materials and Methods

### 3.1. General Experimental Procedures

HPLC was conducted on an Agilent system with a 1260 Quat-Pump and DAD detector. For analytical HPLC, a reverse-phase C18 column (YMC-Pack ODS-A column: 250 mm × 4.6 mm, S-5 μm, 12 nm) was used with a gradient solvent system from 15% to 70% CH_3_CN-H_2_O (0.1% HAc, *v*/*v*), 1.0 mL/min. For semipreparative HPLC, a reverse-phase C18 column (YMC-Pack ODS-A column: 250 mm × 10 mm, S-5 μm, 12 nm) was used with an isocratic solvent system for 30% CH_3_CN-H_2_O (0.1% HAc, *v*/*v*), 1.5 mL/min. UV spectra were acquired with a Thermo Scientific (New York, NY, USA) Evolution 201 UV–visible spectrophotometer. NMR data were collected using an ADVANCE HD 800 MHz and a Bruker Avance III HD 700 MHz spectrometer, where chemical shifts (*δ*) were reported in ppm and referenced to the acetone-*d*_6_ solvent signal (*δ*_H_ 2.05 and *δ*_C_ 29.84, 206.33). ESIMS and MS^2^ were conducted on a 1100-6410 Triple Quad from Agilent (Santa Clara, CA, USA). HRESIMS were conducted on a Thermo (New York, NY, USA) LTQ Orbitrap XL.

### 3.2. Fermentation of Streptomyces *sp.* CPCC 204095

Frozen stock spores of *Streptomyces* sp. CPCC 204095 were thawed, inoculated on culture medium (soluble starch 2.0%, yeast extract 0.4%, malt extract 1.0%, glucose 0.4% and agar 1.5%), and incubated at 28 °C for 7 days for sporulation. Fresh spores were collected and spread on fermentation medium (yeast extract 0.6%, malt extract 2.0%, glucose 1.5%, soybean cake 0.6% and agar 1.5%) plates and incubated at 28 °C for 32–36 h for **1ab** (H_2_ITC) production, or for 20–60 h for monitoring **1ab** (H_2_ITC) and ITC titers.

### 3.3. Extraction and Isolation of Compound ***1ab***

Fermentation culture (5 L) of *Streptomyces* sp. CPCC 204095 was extracted with an equal volume of EtOAc (5% HAc, *v*/*v*) two times. Specifically, each extraction took no longer than a few hours. The combined organic layer was vacuum dried below 30 °C, yielding a dark brown residue (4.85 g). The residue was loaded onto a preparative silica column for fractionation with CH_2_Cl_2_-MeOH (0% MeOH, 20 min; 1% MeOH, 20 min; 2% MeOH, 30 min; 3% MeOH, 60 min, *v*/*v*) at a constant flow rate of 35 mL/min, which yielded 14 fractions from F1-1 to F1-14. Each fraction was analyzed by TLC and HPLC. Fractions from F1-2 to F1-5 were found to contain compound **1ab**.

Fractions from F1-2 to F1-5 were combined and concentrated under reduced pressure below 30 °C, yielding a dark brown residue. The residue was then loaded onto a preparative ODS column for fractionation with MeOH-H_2_O (20% MeOH-H_2_O, 30 min; 25% MeOH-H_2_O, 40 min; 30% MeOH-H_2_O, 15 min; 35% MeOH-H_2_O, 300 min and finally 100% MeOH, 20 min; H_2_O contained 0.1% HAc, *v*/*v*) at a constant flow rate of 25 mL/min, which yielded 72 fractions from F2-1 to F2-72. Each fraction was analyzed by HPLC. Fractions from F2-25 to F2-44 were found to contain compound **1ab**, so they were combined. A part of the preparation was used for semipreparative HPLC (30% MeCN-H_2_O, 1.5 mL/min, t_R_ = 37 min) to obtain the **1ab** sample (2.0 mg) for NMR.

### 3.4. Quantitative Assay of H_2_ITC (***1ab***) and Isatropolone C (ITC)

A freshly prepared **1ab** solution was divided into two parts. One part was vacuum-dried to obtain the quantity of **1ab** in the solution. The other part was serially diluted for analytical HPLC, establishing a linear relationship of **1ab** quantity with its pair peaks area. The linear relationship was used for HPLC assay of **1ab**, or **1ab** titer in *Streptomyces* sp. CPCC 204095. Quantitative assay of isatropolone C (ITC) was also conducted by HPLC in a way similar to H_2_ITC, according to Liu Xiaoyan et al. (Figures S23–S24) [16].

### 3.5. Spontaneous Oxidation of H_2_ITC (***1ab***) to Isatropolone C

(1) H_2_ITC (**1ab**) was dissolved in 3.0 mL 30% MeOH/H_2_O (pH7.0) at a concentration of 0.45 mg/mL. It was equally separated into two 2.0 mL glass vials. One vial was kept at 20 °C, and the other at −20°C. The two vials were then analyzed by HPLC for **1ab** and isatropolone C derived from spontaneous oxidation of **1ab** in 20 and 60 h, respectively. (2) H_2_ITC (**1ab**) was dissolved in 1.0 mL 30% MeOH/H_2_O plus 0.5 mL phosphate buffer (0.1 mol/L, pH8.0) at a concentration of 0.45 mg/mL within a 2.0 mL glass vial. It was kept at 20 °C, and then analyzed by HPLC for **1ab** and isatropolone C derived from spontaneous oxidation of **1ab** in 1.0 and 40.0 min, respectively.

## Figures and Tables

**Table 1:** NMR data of 7,12-dihydroisatropolone C (**1ab**) and isatropolone C in acetone-*d*_6_.

	7,12-Dihydroisatropolone C (**1ab**)			Isatropolone C	
Position	*δ*_C_, Type	*δ*_H_, Mult, (*J* in Hz)	HMBC	*δ*_C_, Type	*δ*_H_, Mult, (*J* in Hz)
1	10.20, 10.11, CH_3_	0.92, t (7.3)	C-2, C-3	10.11, CH_3_	0.95, t
2	28.30, 28.01, CH_2_	1.35, overlap	C-3, C-4	28.18, CH_2_	1.39, m
3	76.43, 76.34, CH	4.78, m; 4.70, m * (3.5, 7.5)	C-1, C-4	76.62, CH	4.84, m *
3-OH					
4	199.06, 198.99, C			199.77, C	
6	188.12, 187.41, C			190.21, C	
7	42.44, 42.33, CH_2_	2.91, d; 2.74, d (15.1)	C-8, C-9, C-12	134.35, CH_2_	7.12, s
8	184.18, 182.76, C			185.09, C	
9	131.68, 131.43, C			132.02, C	
10	158.96, 156.82, C			159.47, C	
11	142.08, 141.98, C			144.83, C	
12	40.48, 40.37, CH	3.65, dd; 3.56, dd (3.0, 15.1)	C-7	135.54, CH	
13	122.58, 119.85, C			120.98, C	
14	162.97, 162.89, C			162.58, C	
15	106.95, 106.90, C	7.70, s; 7.68, s	C-14, C-16, C-17	107.57, C	7.77, s
16	168.41, 168.39, C			170.24, C	
17	20.77, 20.74, CH_3_	2.58, s	C-15, C-16	21.17, CH_3_	2.64, s
1′	110.14, 110.08, CH	5.69, s; 5.63, s	C-3′, C-5′	109.89, CH	5.78, s
2′	81.54, 81.37, C			82.04, C	
2′-OH					
3′	69.20, 68.91, CH	4.81, d; 4.62, d *		68.79, CH	5.00, brs *
3′-OH					
4′	80.75, 80.39, CH	3.33, m; 3.32, m	C-3′, C-5′, C-7′	79.96, CH	3.34, brs
5′	67.93, 66.65, CH	4.24, m; 4.08, m		65.98, CH	4.15, m
6′	18.31, 18.26, CH_3_	1.29, d; 1.28, d (6.4)	C-4′, C-5′,	18.75, CH_3_	1.31, d
7′	57.47, 57.33, CH_3_	3.37, s	C-4′	57.90, CH_3_	3.34, s

* Exchangeable.

## Data Availability

Not applicable.

## Supplementary Materials

The following supporting information can be downloaded at: www.mdpi.com/article/10.3390/molecules27092882/s1. Figure S1. Three HPLC chromatograms of EtOAc extract (with an extraction time of 2 h) of *Streptomyces* sp. CPCC 204095. Figure S2. A parallel HPLC analysis of *Streptomyces* sp. CPCC 204095 revealing a relationship of the pair peaks with isatropolone C peak. Figure S3. HPLC separation of the pair peaks and confirmation of their exchange. Figure S4. HPLC of freshly prepared compound **1ab**. Figure S5. UV–visible absorption of compound **1ab**. Figure S6. LC-MS of compound **1ab** sample containing a small amount of isatropolone C. Figure S7. HRESIMS of compound **1ab**. Figure S8. Alignment of ^13^C NMR spectra of 7,12-dihydroisatropolone C (**1ab**) and isatropolone C. Figure S9. ^1^H NMR spectrum (700 MHz) of 7,12-dihydroisatropolone C (**1ab**) in acetone-*d*_6_. Figure S10. ^13^C NMR spectrum (700 MHz) of 7,12-dihydroisatropolone C (**1ab**) in acetone-*d*_6_. Figure S11. DEPT spectrum (700 MHz) of 7,12-dihydroisatropolone C (**1ab**) in acetone-*d*_6_. Figure S12. ^1^H-^1^H COSY spectrum (800 MHz) of 7,12-dihydroisatropolone C (**1ab**) in acetone-*d*_6_. Figure S13. HSQC spectrum (800 MHz) of 7,12-dihydroisatropolone C (**1ab**) in acetone-*d*_6_. Figure S14. HMBC spectrum (800 MHz) of 7,12-dihydroisatropolone C (**1ab**) in acetone-*d*_6_. Figure S15. ^13^C-NMR spectrum (0–30 ppm) of 7,12-dihydroisatropolone C (**1ab**). Figure S16. ^13^C-NMR spectrum (30–60 ppm) of 7,12-dihydroisatropolone C (**1ab**). Figure S17. ^13^C-NMR spectrum (60–90 ppm) of 7,12-dihydroisatropolone C (**1ab**). Figure S18. ^13^C-NMR spectrum (90–120 ppm) of 7,12-dihydroisatropolone C (**1ab**). Figure S19. ^13^C-NMR spectrum (120–150 ppm) of 7,12-dihydroisatropolone C (**1ab**). Figure S20. ^13^C-NMR spectrum (150–180 ppm) of 7,12-dihydroisatropolone C (**1ab**). Figure S21. ^13^C-NMR spectrum (180–210 ppm) of 7,12-dihydroisatropolone C (**1ab**). Figure S22. Active hydrogen atom signals in the ^1^H-NMR spectrum (10–12 ppm) of 7,12-dihydroisatropolone C (**1ab**). Figure S23. HPLC for an identical amount of H_2_ITC and ITC. Figure S24. Analytical HPLC of various amounts of H_2_ITC. Figure S25. HPLC of 7,12-dihydroisatropolone C (in 30% MeOH) changing to isatropolone C at pH7.0 plus 20 °C. Figure S26. HPLC of 7,12-dihydroisatropolone C (in 30% MeOH) changing to isatropolone C at pH8.0 plus 20 °C. Figure S27. HPLC of 7,12-dihydroisatropolone C (in 30% MeOH) changing to isatropolone C at pH7.0 plus −20 °C. Figure S28. H_2_ITC conjugates NH_3_ for 7,12-dihydroisarubrolone C production. Scheme S1. Biosynthesis of rubrulone A focusing on oxidative rearrangement for tropolone-ring construction from mono-cyclic/aromatic intermediate proposed by Yan et al. (route 1), and biosynthesis of isatropolone C (ITC) focusing on oxidative rearrangement for tropolone-ring construction from bi-cyclic/aromatic intermediate proposed by Cai et al. (route 2). Figure S29. Two compounds **9**–**10** characterized from *S. lividans* heterologously expressing *istG-R* for the aglycone biosynthesis of isatropolone (reported by Cai et al.).
