# Supplementary material for: Dihydroisatropolone C from Streptomyces and Its Implication in Tropolone-Ring Construction for Isatropolone Biosynthesis
Source: Molecules. 2022 Apr 30;27(9):2882. doi: 10.3390/molecules27092882 (PMC9099902; doi:10.3390/molecules27092882)
Supplement: Supplementary file 1 [file molecules-27-02882-s001.zip › molecules-1689923-supplementary.pdf]

Supplementary Information

Dihydroisatropolone C from *Streptomyces* and its implication in tropolone-ring construction for isatropolones biosynthesis

Jiachang Liu, Xiaoyan Liu, Jie Fu, Bingya Jiang, Shufen Li*, Linzhuan Wu*

NHC Key Laboratory of Biotechnology of Antibiotics, CAMS Key Laboratory of Synthetic Biology for Drug Innovation, Institute of Medicinal Biotechnology, Chinese Academy of Medical Sciences & Peking Union Medical College, Tiantan Xili, Beijing 100050, China

* **Shufen Li** - Institute of Medicinal Biotechnology, Chinese Academy of Medical Sciences and Peking Union Medical College, Beijing 100050, P. R. China;
Email: lisf0229@163.com
* **Linzhuan Wu** - Institute of Medicinal Biotechnology, Chinese Academy of Medical Sciences and Peking Union Medical College, Beijing 100050, P. R. China;
Email: wulinzhuan@imb.pumc.edu.cn

Table of Contents

Figure S1. Three HPLC chromatograms of EtOAc extract (with an extraction time of 2 hours) of *Streptomyces* sp. CPCC 204095 .... S4

Figure S2. A parallel HPLC analysis of *Streptomyces* sp. CPCC 204095 revealing a relationship of the pair peaks with isatropolone C peak S5

Figure S3. HPLC separation of the pair peaks and confirmation of their exchange S6

Figure S4. HPLC of freshly prepared compound **1ab**. S7

Figure S5. UV-visible absorption of compound **1ab** S7

Figure S6. LC-MS of compound **1ab** sample conaining a small amount of isatropolone C. S8

Figure S7. HRESIMS of compound **1ab** S9

Figure S8. Alignment of ^{13}C NMR spectra of 7,12-dihydroisatropolone C (**1ab**) and isatropolone C. S9

Figure S9. ^{1}H NMR spectrum (700 MHz) of 7,12-dihydroisatropolone C (**1ab**) in acetone-d_6 .... S10

Figure S10. ^{13}C NMR spectrum (700 MHz) of 7,12-dihydroisatropolone C (**1ab**) in acetone-d_6 .... S10

Figure S11. DEPT spectrum (700 MHz) of 7,12-dihydroisatropolone C (**1ab**) in acetone-d_6 .... S11

Figure S12. ^{1}H-^{1}H COSY spectrum (800 MHz) of 7,12-dihydroisatropolone C (**1ab**) in acetone-d_6 .... S11

Figure S13. HSQC spectrum (800 MHz) of 7,12-dihydroisatropolone C (**1ab**) in acetone-d_6 .... S12

Figure S14. HMBC spectrum (800 MHz) of 7,12-dihydroisatropolone C (**1ab**) in acetone-d_6 .... S12

Figure S15. ^{13}C-NMR spectrum (0-30 ppm) of 7,12-dihydroisatropolone C (**1ab**) S13

Figure S16. ^{13}C-NMR spectrum (30-60 ppm) of 7,12-dihydroisatropolone C (**1ab**) S13

Figure S17. ^{13}C-NMR spectrum (60-90 ppm) of 7,12-dihydroisatropolone C (**1ab**) S14

Figure S18. ^{13}C-NMR spectrum (90-120 ppm) of 7,12-dihydroisatropolone C (**1ab**) S14

Figure S19. ^{13}C-NMR spectrum (120-150 ppm) of 7,12-dihydroisatropolone C (**1ab**) S15

Figure S20. ^{13}C-NMR spectrum (150-180 ppm) of 7,12-dihydroisatropolone C (**1ab**) S15

Figure S21. ^{13}C-NMR spectrum (180-210 ppm) of 7,12-dihydroisatropolone C (**1ab**) S16

Figure S22. Active hydrogen atoms signals in the ^{1}H-NMR spectrum (10-12 ppm) of 7,12-dihydroisatropolone C (**1ab**). S16

Figure S23. HPLC for an identical amount of H_2ITC and ITC. S17

Figure S24. Analytical HPLC of various amounts of H_2ITC S18

Figure S25. HPLC of 7,12-dihydroisatropolone C (in 30% MeOH) changing to isatropolone C at pH7.0 plus 20°C. S19

Figure S26. HPLC of 7,12-dihydroisatropolone C (in 30% MeOH) changing to isatropolone C at pH8.0 plus 20°C. S19

Figure S27. HPLC of 7,12-dihydroisatropolone C (in 30% MeOH) changing to isatropolone C at pH7.0 plus −20°C. S19

Figure S28. H_2ITC conjugates NH_3 for 7,12-dihydroisarubrolone C production. S20

Scheme S1. Biosynthesis of rubrulone A focusing on oxidative rearrangement for tropolone-ring construction from mono-cyclic/aromatic intermediate proposed by Yan *et al* (route 1), and biosynthesis of isatropolone C (ITC) focusing on oxidative rearrangement for tropolone-ring construction from bi-cyclic/aromatic intermediate proposed by Cai *et al* (route 2). S21

Figure S29. Two compounds **9-10** characterized from *S. lividans* heterologous-expressing *istG-R* for the aglycone biosynthesis of isatropolone (reported by Cai *et al.*) S22

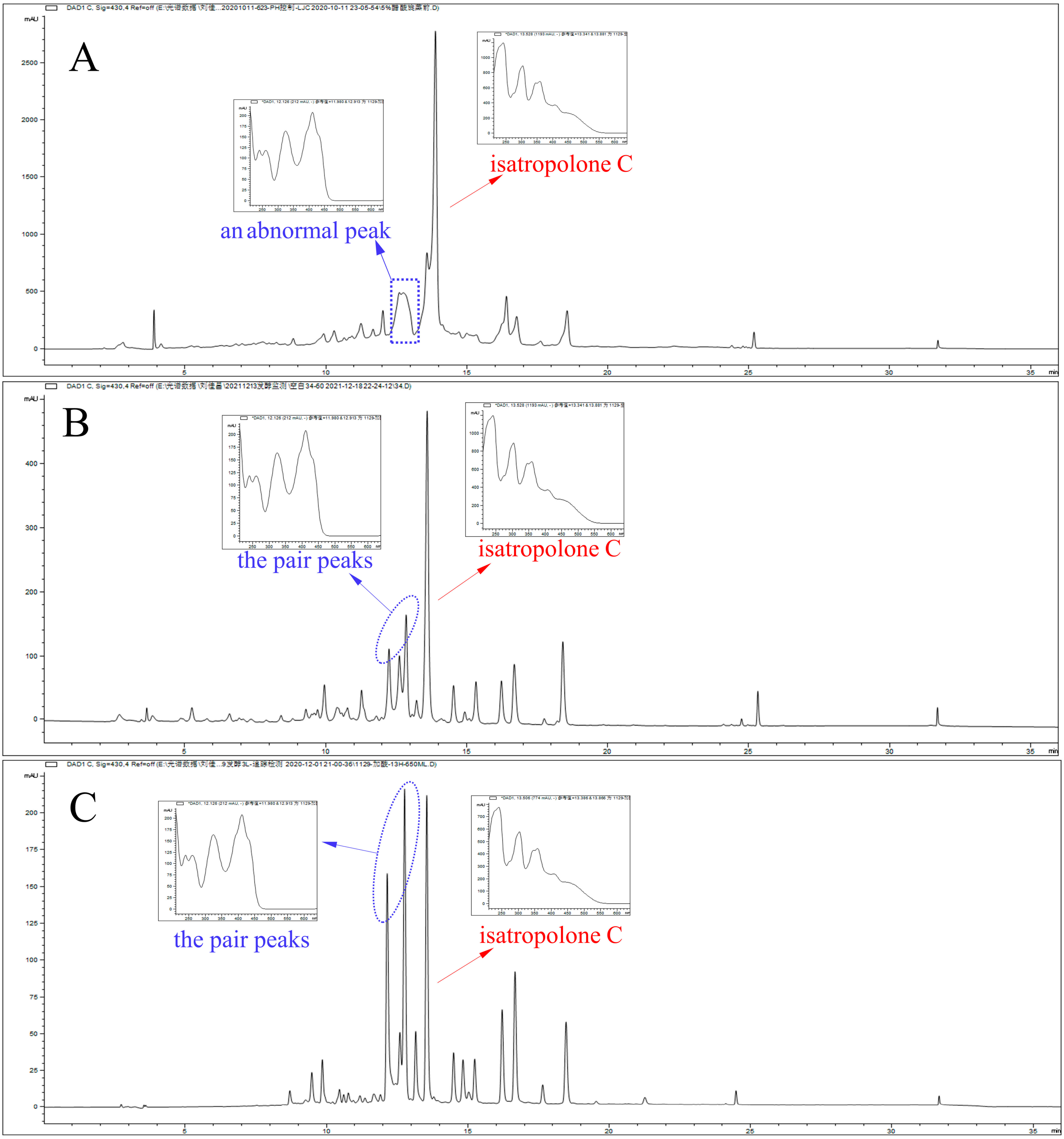

Figure S1. Three HPLC chromatograms of EtOAc extract (with an extraction time of 2 hours) of *Streptomyces* sp. CPCC 204095

(A) EtOAc extract, Shiseido Capcell Pack C18 AQ column (4.6 mm × 250 mm, 5 µm).

(B) EtOAc extract, YMC-Pack ODS-A column (250 mm × 4.6 mm, S-5 µm, 12 nm).

(C) Acidified (5% HAc) EtOAc extract, YMC-Pack ODS-A column (250 mm × 4.6 mm, S-5 µm, 12 nm).

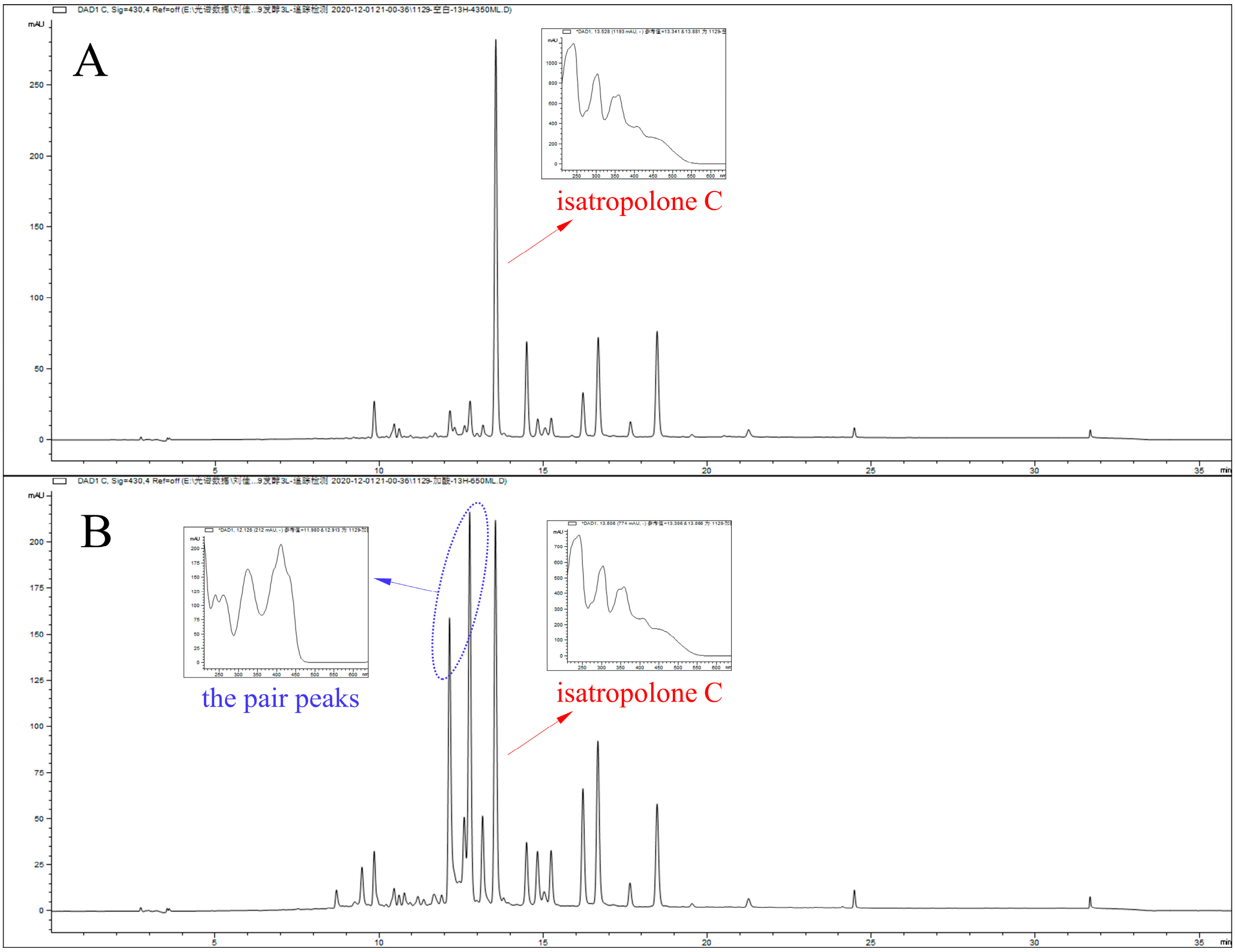

Figure S2. A parallel HPLC analysis of *Streptomyces* sp. CPCC 204095 revealing a relationship of the pair peaks with isatropolone C peak

(A) EtOAc extract (with an extraction time of 24 hours);

(B) acidified (5% HAc) EtOAc extract (with an extraction time of 2 hours).

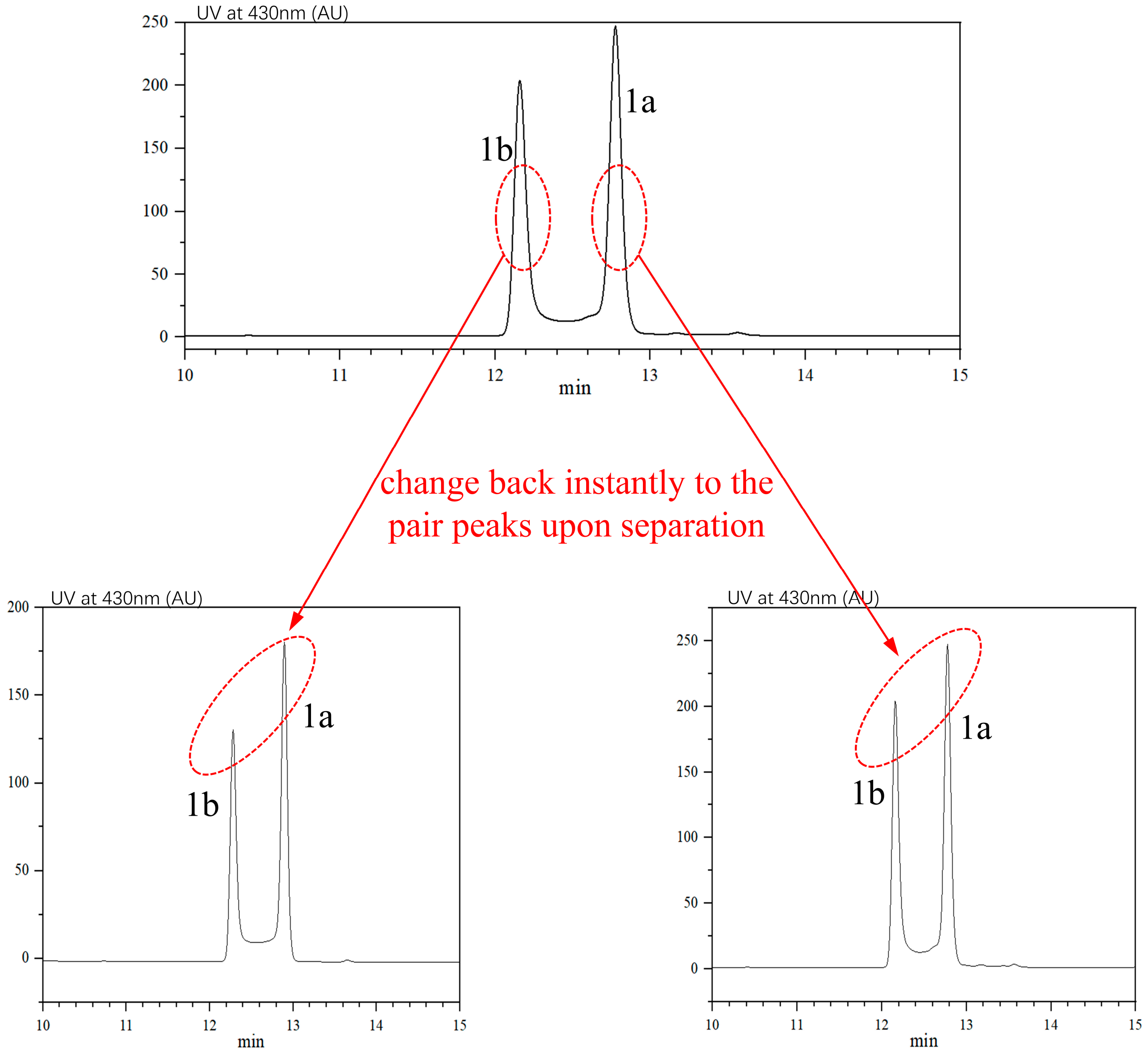

Figure S3. HPLC separation of the pair peaks and confirmation of their exchange

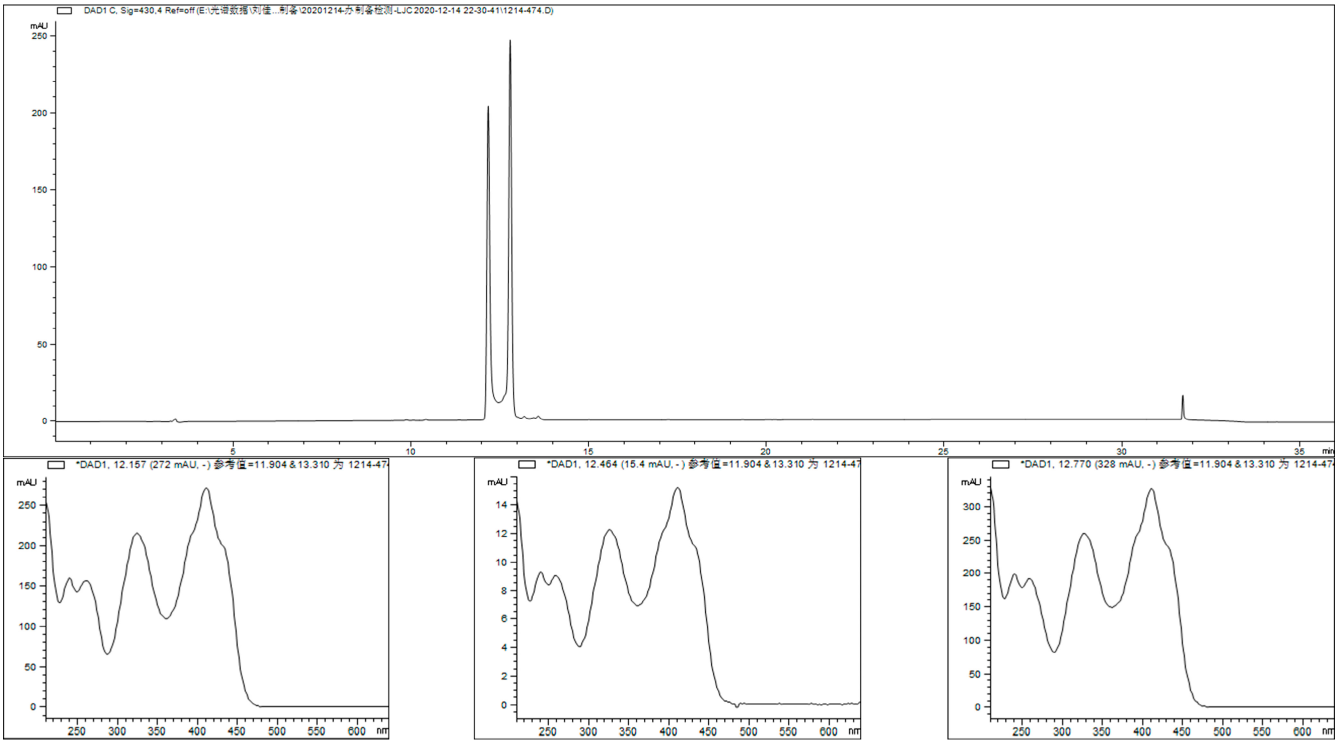

Figure S4. HPLC of freshly prepared compound **1ab**.

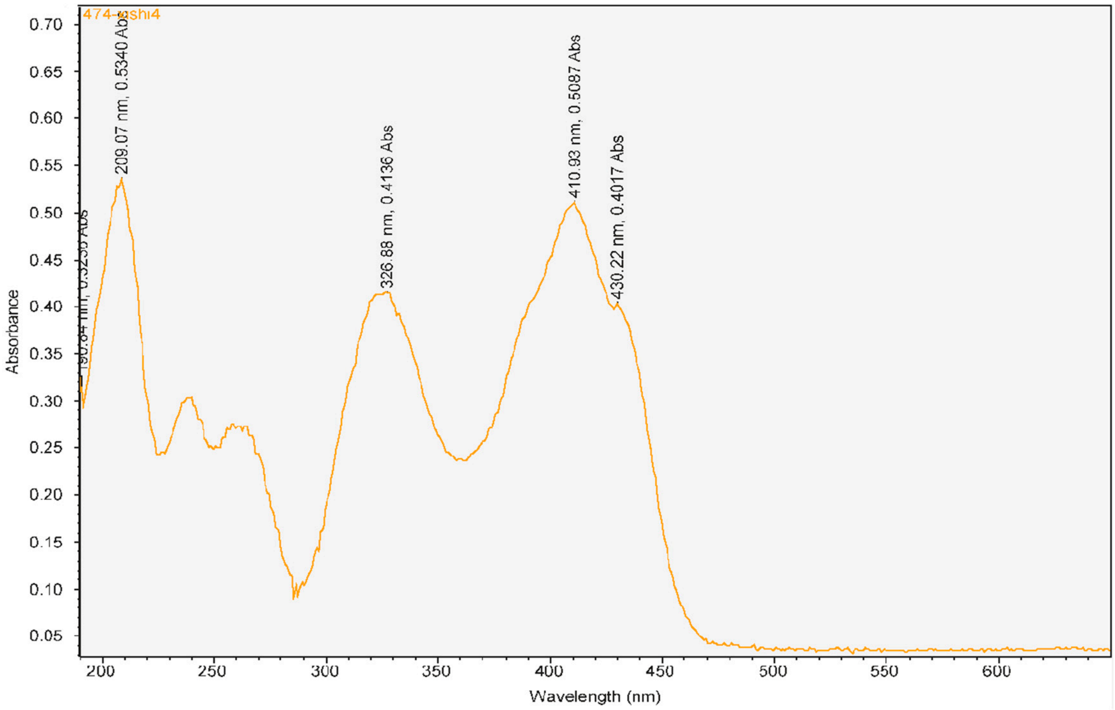

Figure S5. UV-visible absorption of compound **1ab**

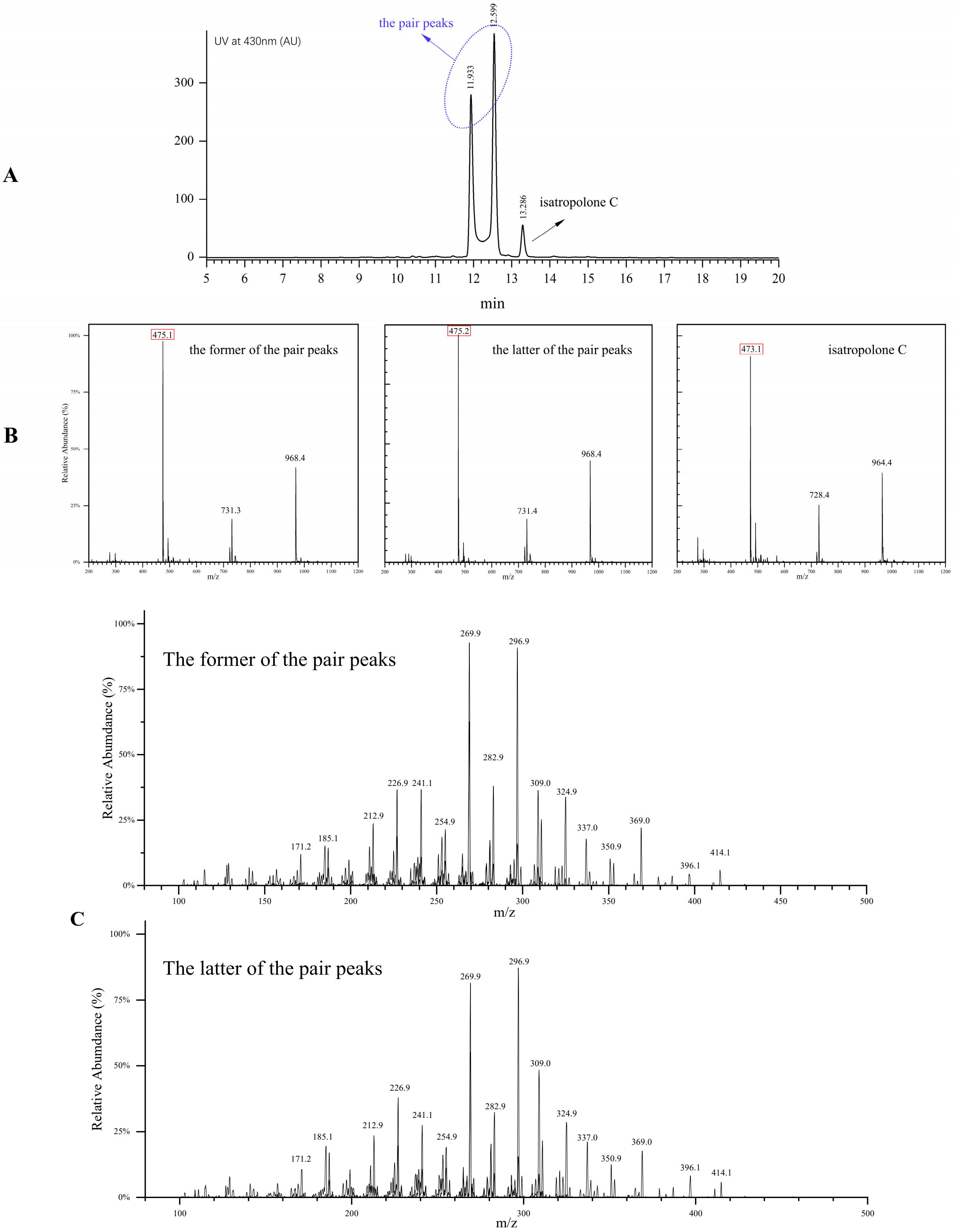

Figure S6. LC-MS of compound **1ab** sample conaining a small amount of isatropolone C.

(A) HPLC of the pair peaks and isatropolone C.

(B) MS of the pair peaks and isatropolone C.

(C) MS^2 of the pair peaks.

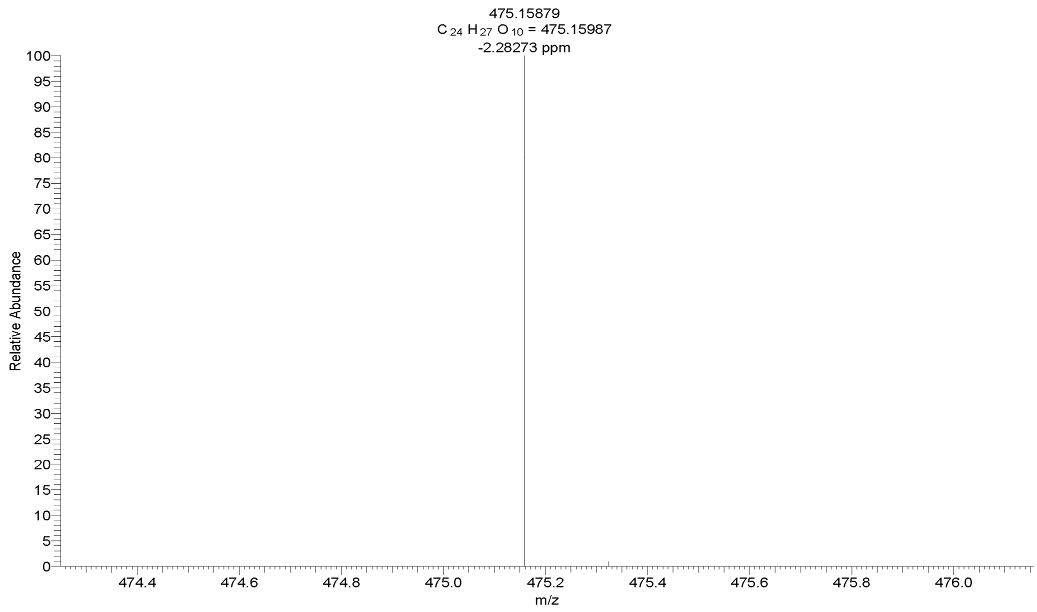

Figure S7. HRESIMS of compound **1ab**

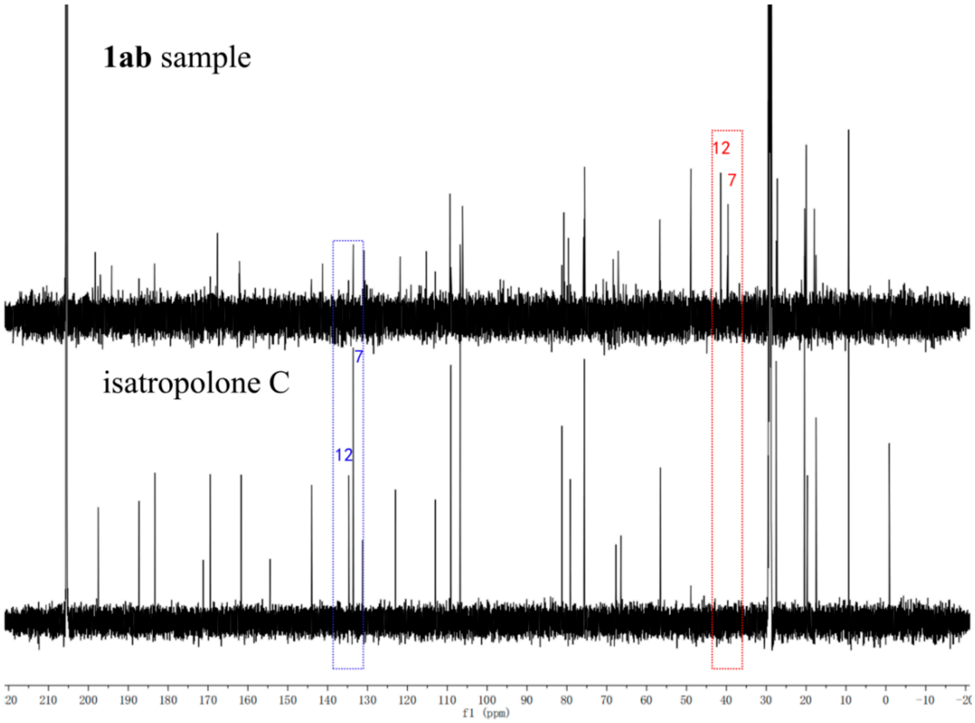

Figure S8. Alignment of ^{13}C NMR spectra of 7,12-dihydroisatropolone C (**1ab**) and isatropolone C.

Signals for C-7 and C-12 were enclosed in dotted rectangle.

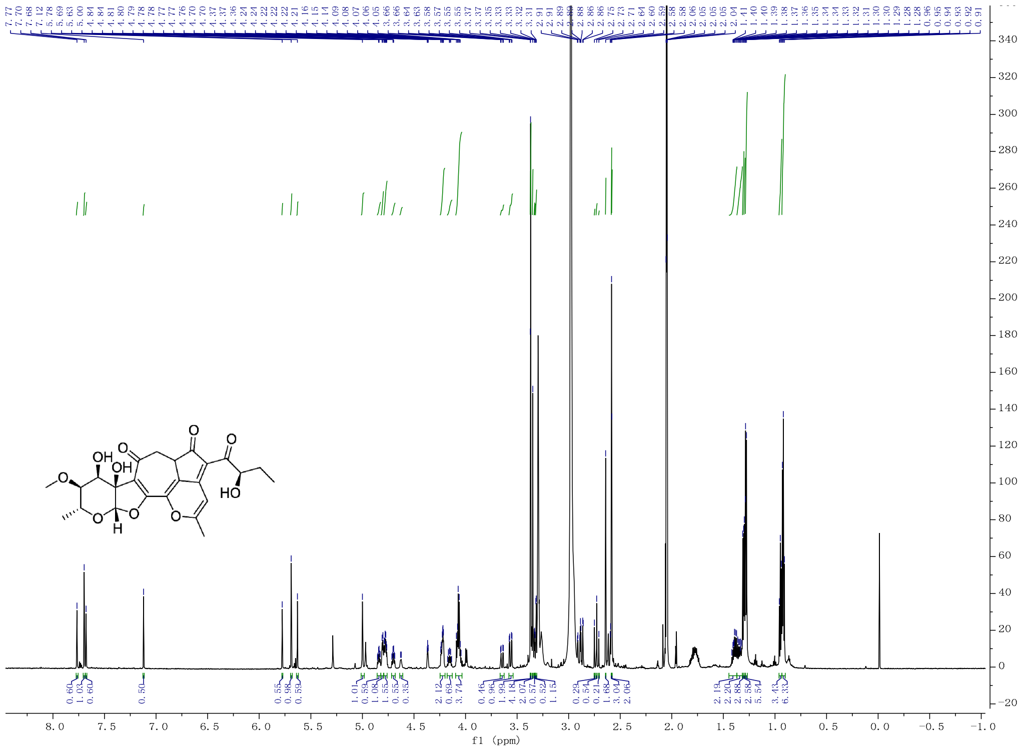

Figure S9. ^{1}H NMR spectrum (700 MHz) of 7,12-dihydroisatropolone C (**1ab**) in acetone-d_6

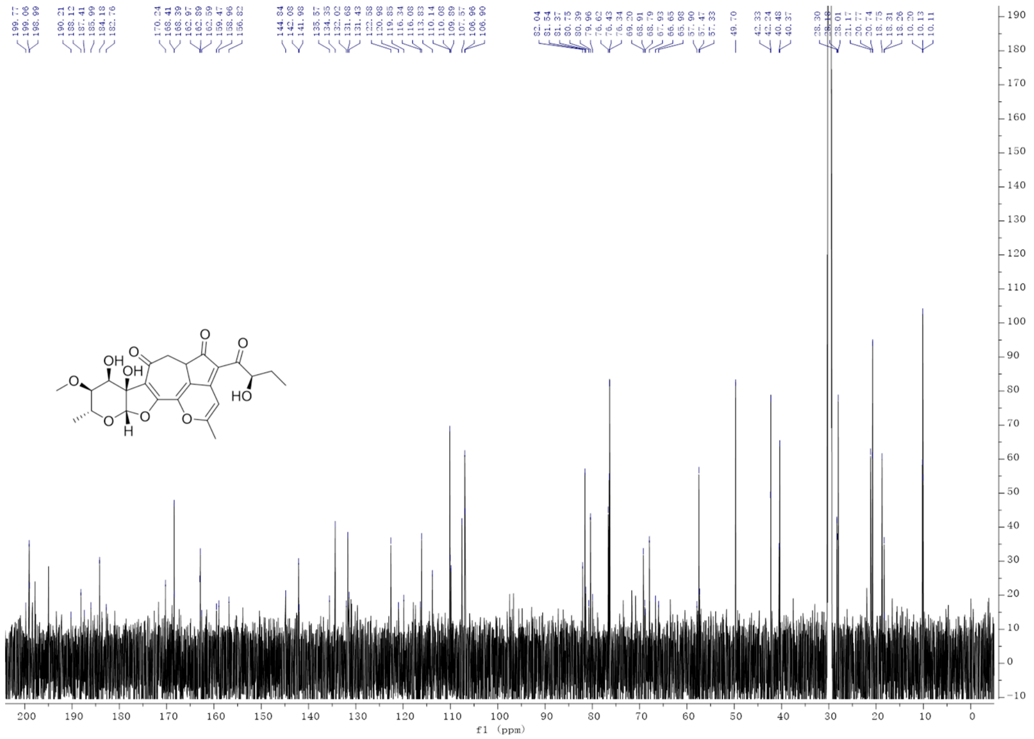

Figure S10. ^{13}C NMR spectrum (700 MHz) of 7,12-dihydroisatropolone C (**1ab**) in acetone-d_6

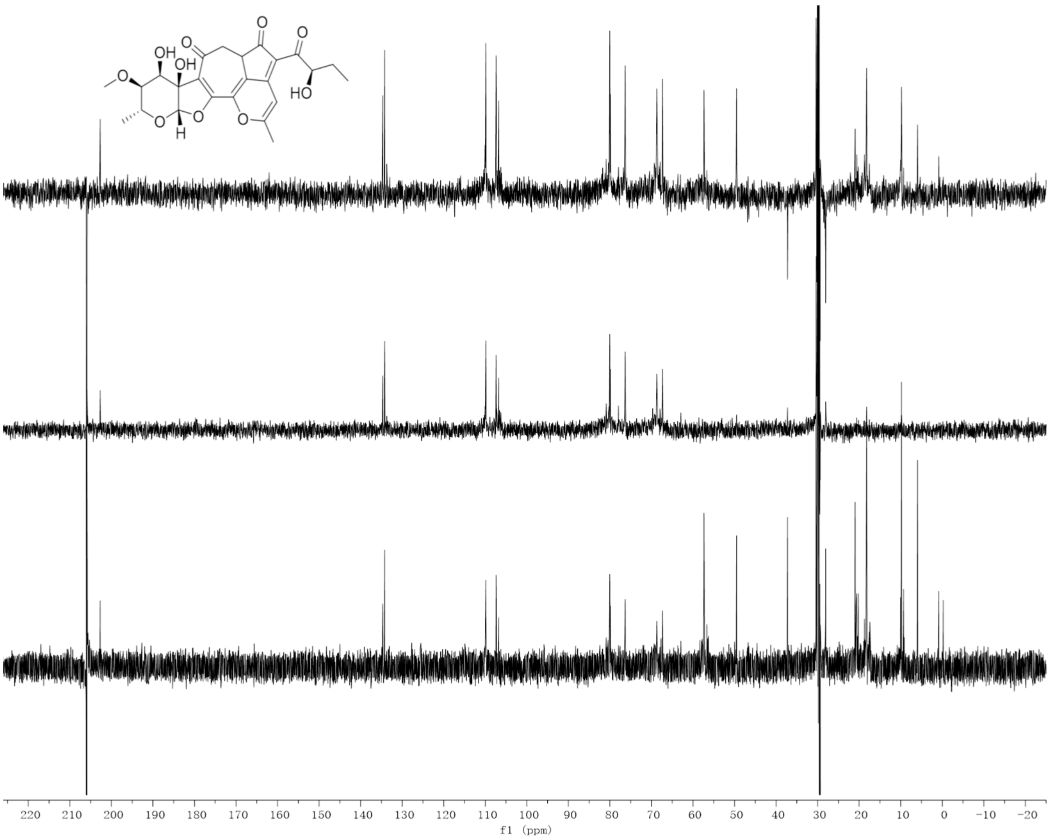

Figure S11. DEPT spectrum (700 MHz) of 7,12-dihydroisatropolone C (**1ab**) in acetone-d_6

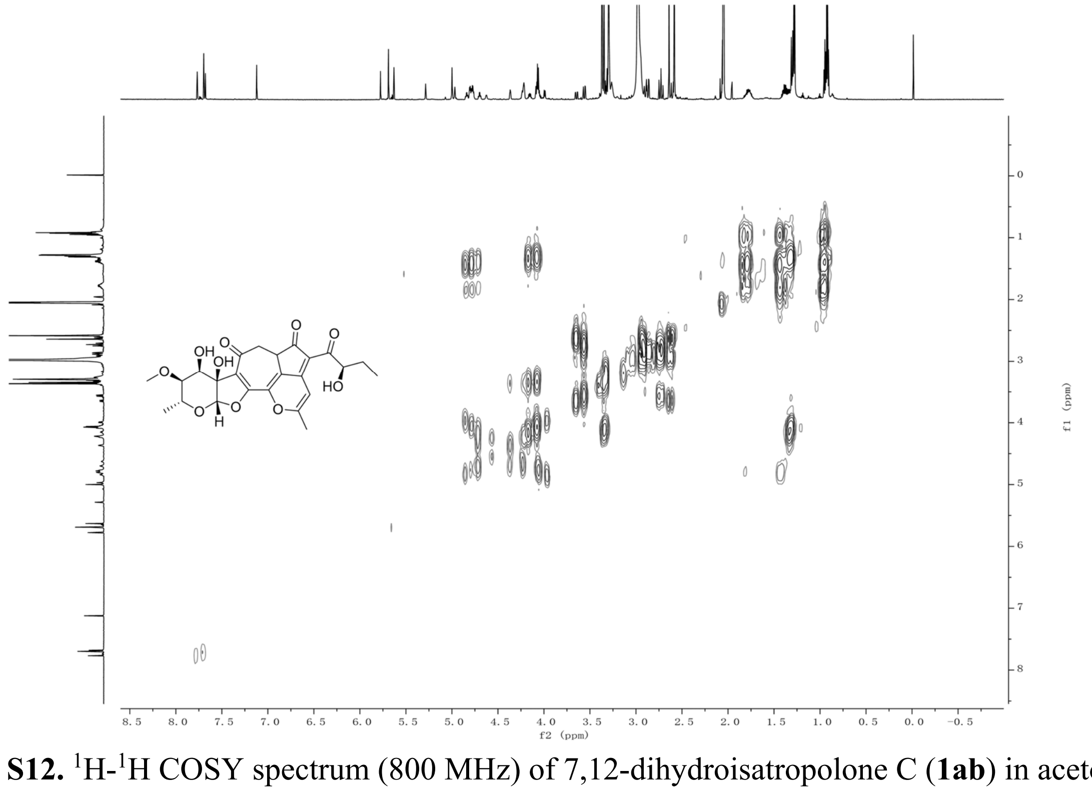

Figure S12. ^{1}H-^{1}H COSY spectrum (800 MHz) of 7,12-dihydroisatropolone C (**1ab**) in acetone-d_6

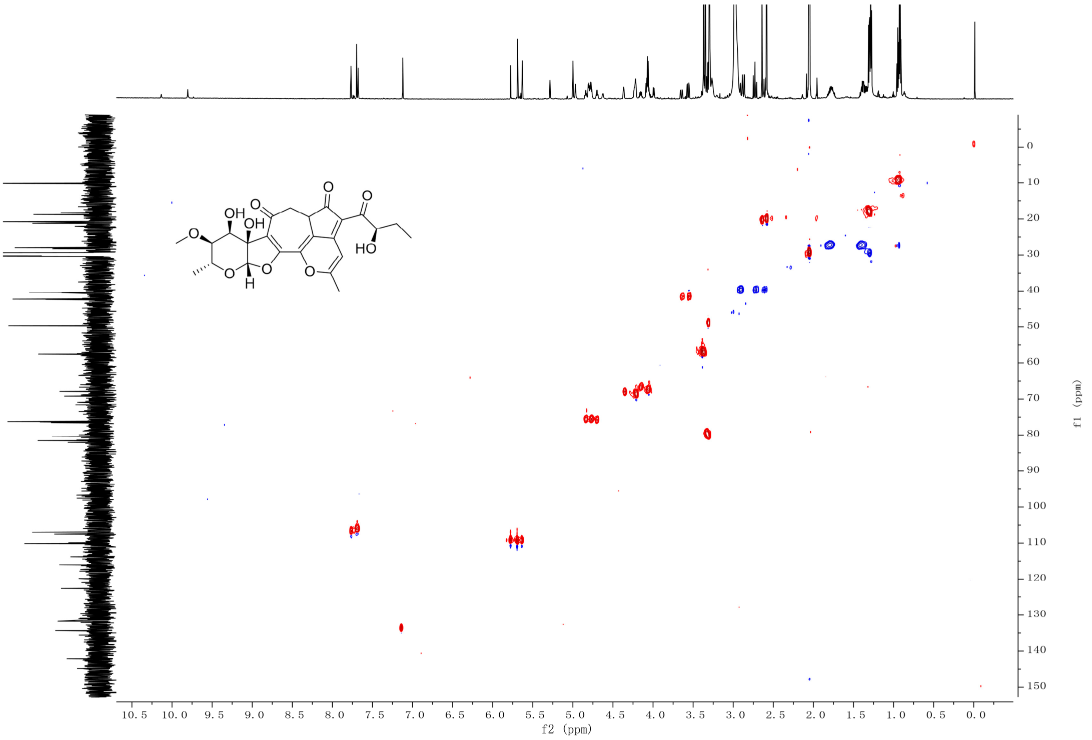

Figure S13. HSQC spectrum (800 MHz) of 7,12-dihydroisatropolone C (**1ab**) in acetone-d_6

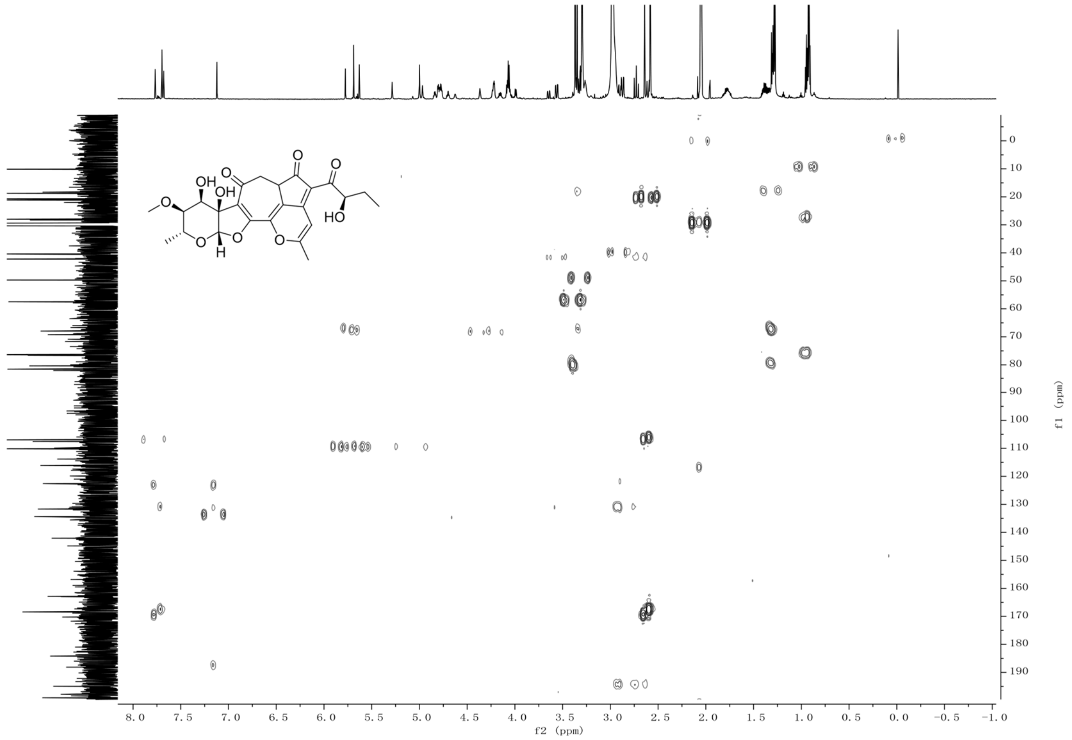

Figure S14. HMBC spectrum (800 MHz) of 7,12-dihydroisatropolone C (**1ab**) in acetone-d_6

The ^{13}C-NMR spectrum of 7,12-dihydroisatropolone C (**1ab**) in Figure S7 was enlarged and separated as the following Figure S15-S21 to view pair signals.

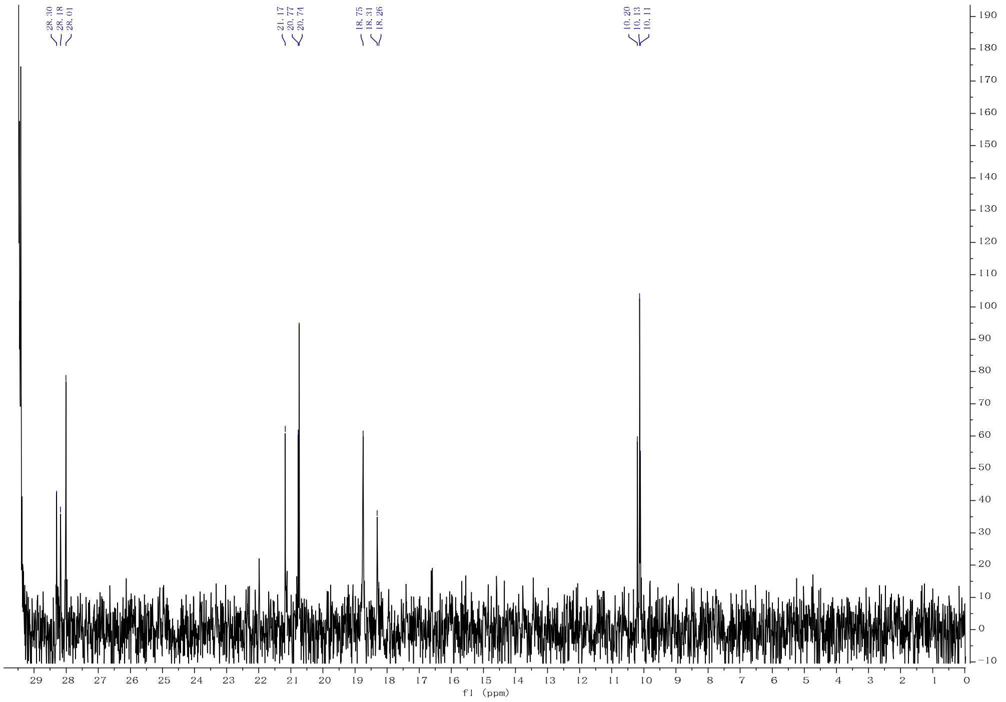

Figure S15. ^{13}C-NMR spectrum (0-30 ppm) of 7,12-dihydroisatropolone C (**1ab**)

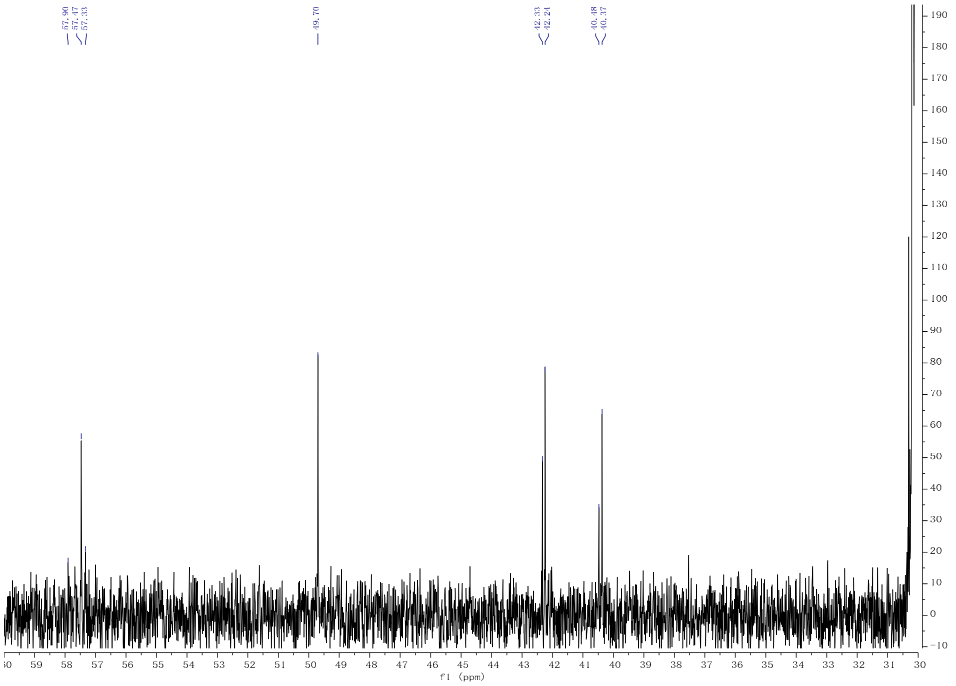

Figure S16. ^{13}C-NMR spectrum (30-60 ppm) of 7,12-dihydroisatropolone C (**1ab**)

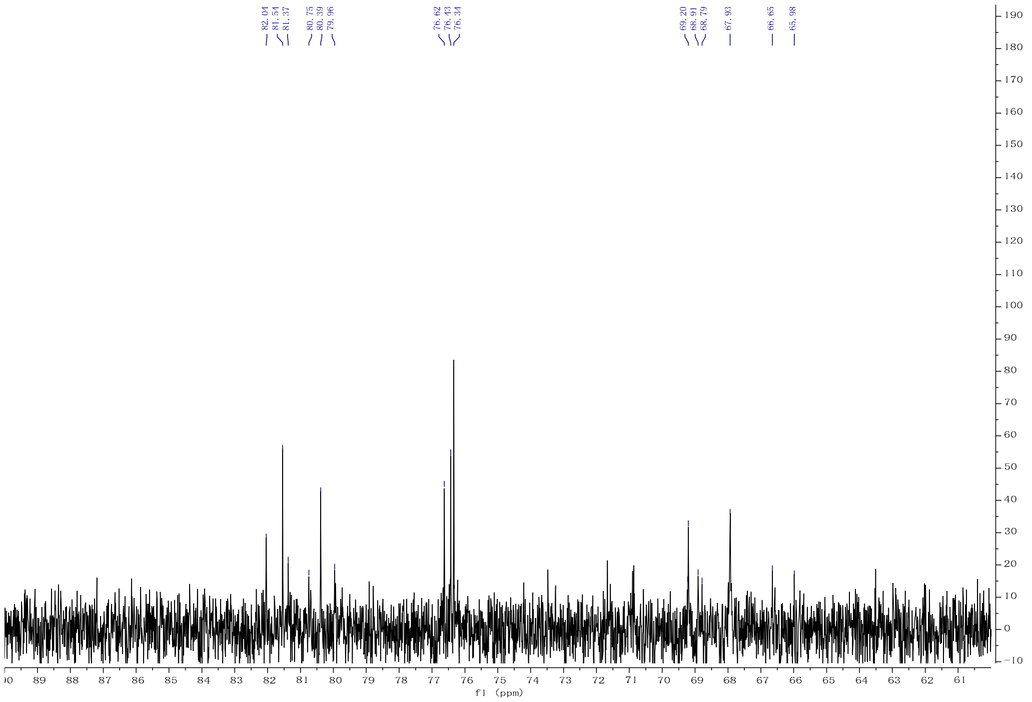

Figure S17. ^{13}C-NMR spectrum (60-90 ppm) of 7,12-dihydroisatropolone C (**1ab**)

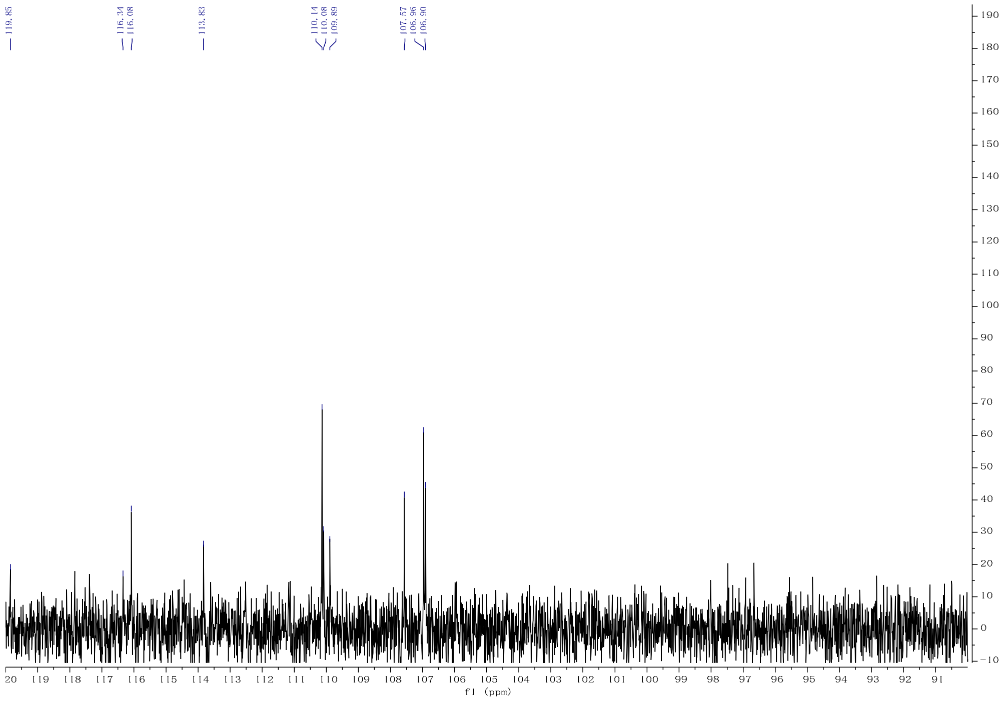

Figure S18. ^{13}C-NMR spectrum (90-120 ppm) of 7,12-dihydroisatropolone C (**1ab**)

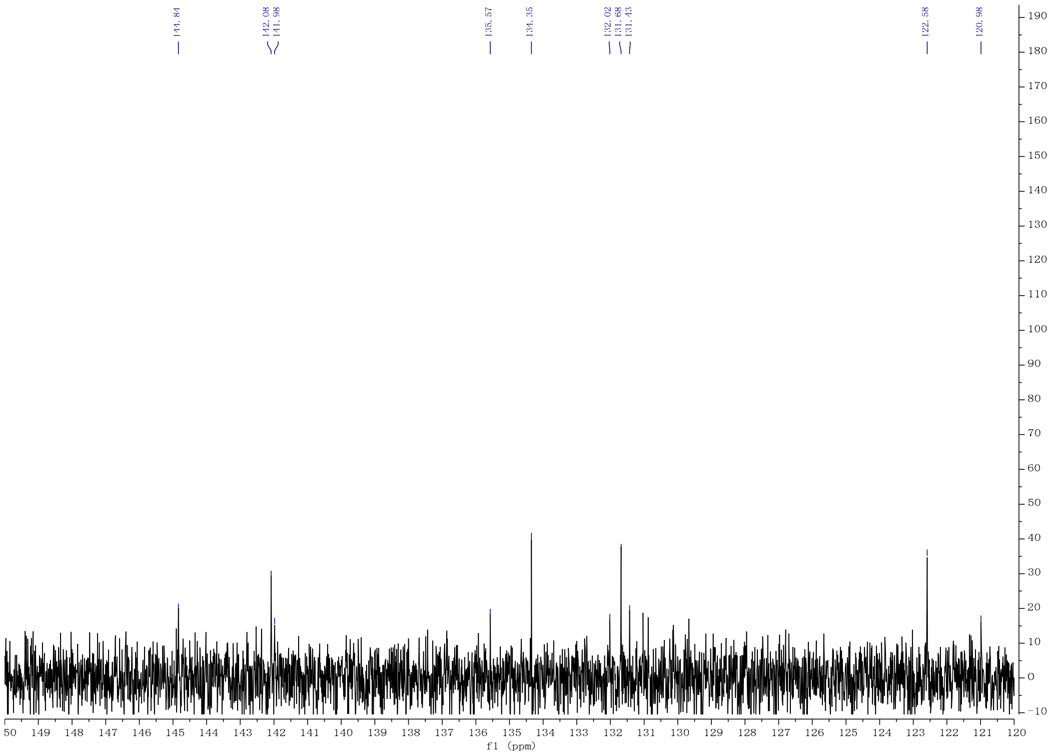

Figure S19. ^{13}C-NMR spectrum (120-150 ppm) of 7,12-dihydroisatropolone C (**1ab**)

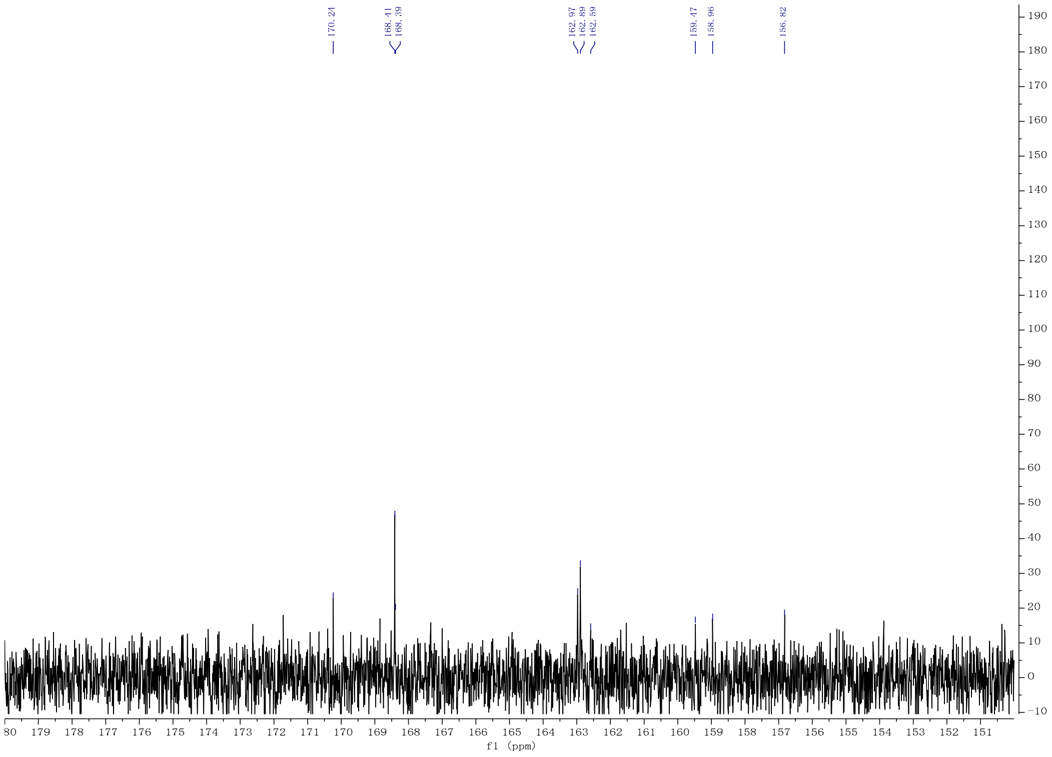

Figure S20. ^{13}C-NMR spectrum (150-180 ppm) of 7,12-dihydroisatropolone C (**1ab**)

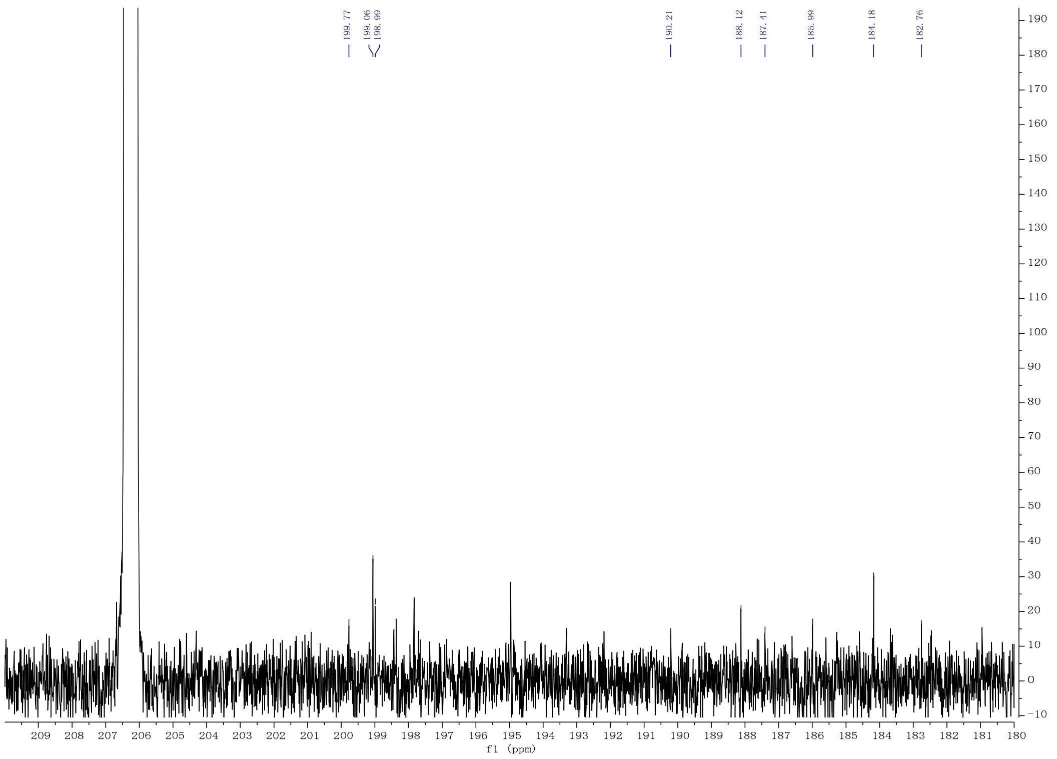

Figure S21. ^{13}C-NMR spectrum (180-210 ppm) of 7,12-dihydroisatropolone C (**1ab**)

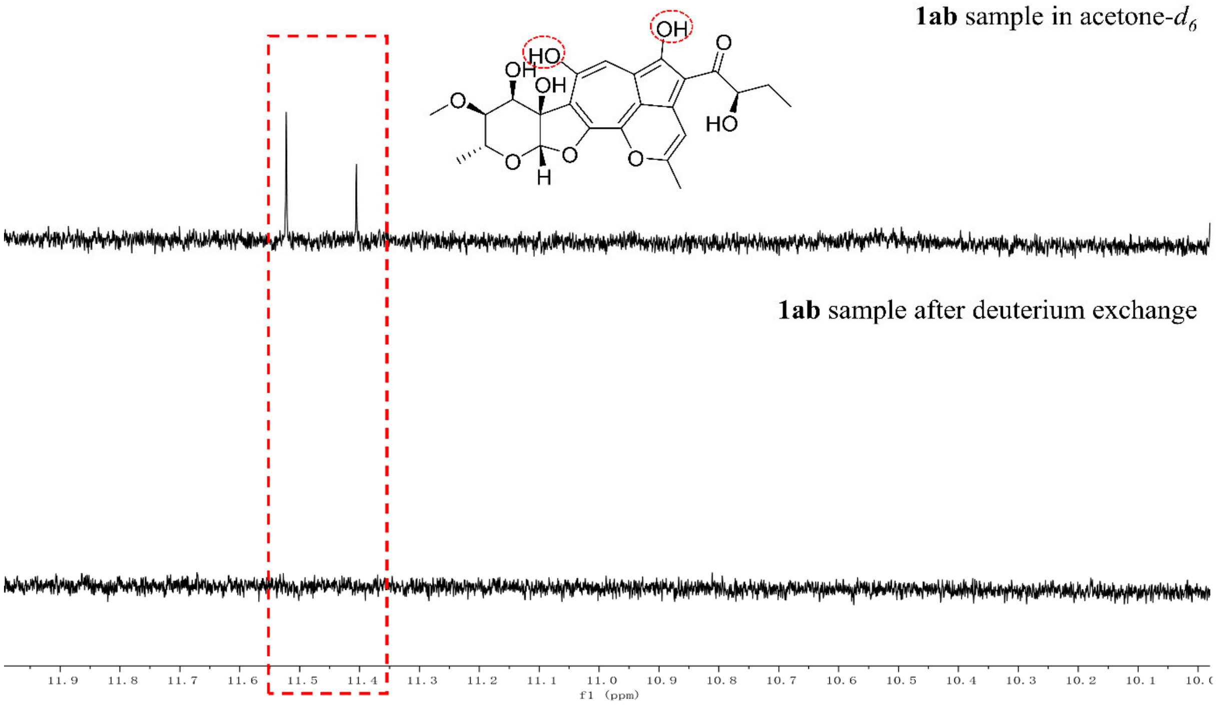

Figure S22. Active hydrogen atoms signals in the ^{1}H-NMR spectrum (10-12 ppm) of 7,12-dihydroisatropolone C (**1ab**).

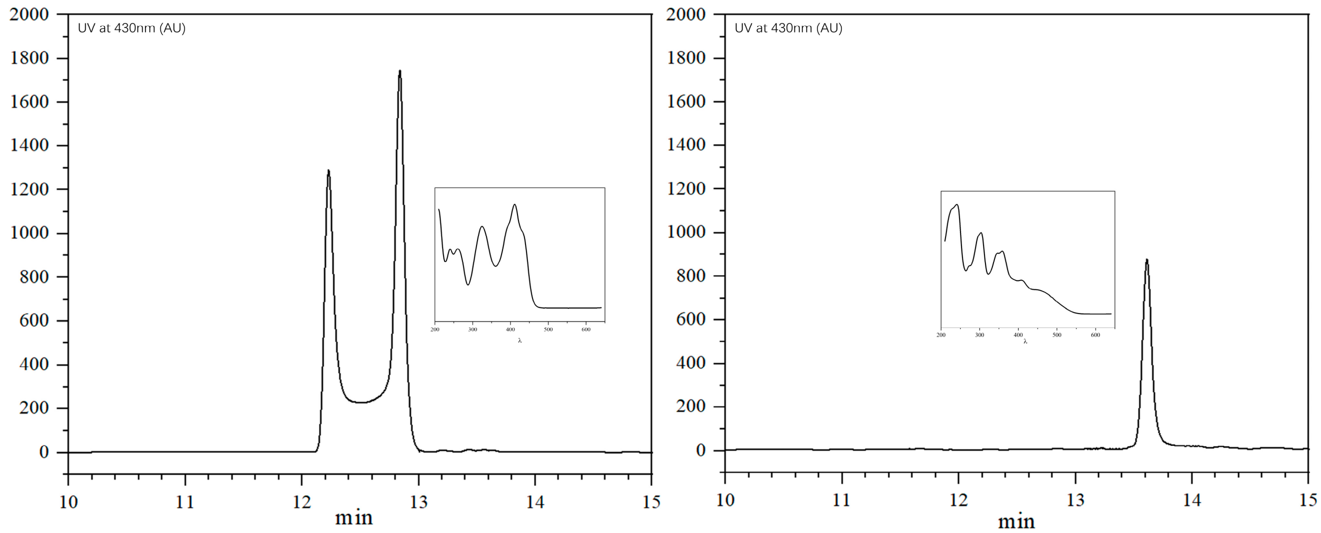

Figure S23. HPLC for an identical amount of H_2ITC and ITC.

For analytical HPLC, a reverse-phase C18 column (YMC-Pack ODS-A column: 250 mm × 4.6 mm, S-5 μm, 12 nm) was used with a gradient solvent system from 15% to 70% CH_3CN-H_2O (0.1% HAc, v/v), 1.0 mL/min. The injection volume is 30 μl for each sample.

The H_2ITC pair peaks area is 3.82 times greater than that of ITC at 430 nm for an identical amount of H_2ITC and ITC.

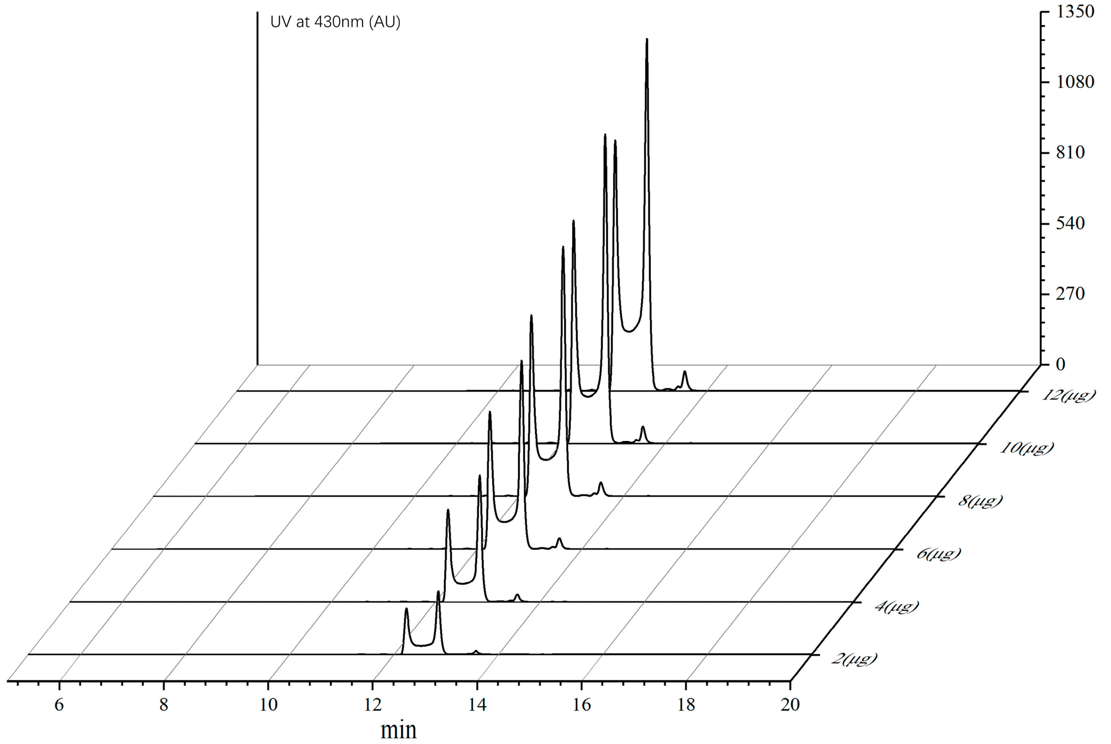

Figure S24. Analytical HPLC of various amounts of H_2ITC

For analytical HPLC, a reverse-phase C18 column (YMC-Pack ODS-A column: 250 mm × 4.6 mm, S-5 μm, 12 nm) was used with a gradient solvent system from 15% to 70% CH_3CN-H_2O (0.1% HAc, v/v), 1.0 mL/min. The injection volume is 20 μl for each sample.

H_2ITC (μg)	H_2ITC pair peaks area	ITC peak area
2	3509.1	88.1
4	6110.4	161.0
6	10518.1	233.3
8	13997	304.1
10	17439.9	369.7
12	20654.5	428.2

A linear relationship was thus established for H_2ITC with its HPLC pair peaks area.
y = 1759.9x - 281.27; R^2 = 0.9971; y refers to H_2ITC pair peaks area, x refers to H_2ITC (μg).
Note: There are about 10% ITC in the H_2ITC samples, according to ITC peak area (after normalization by peak area ratio for identical amounts of H_2ITC and ITC from Figure S22). ITC peak area is not taken into account in establishing the linear relationship of H_2ITC with its pair peaks area.

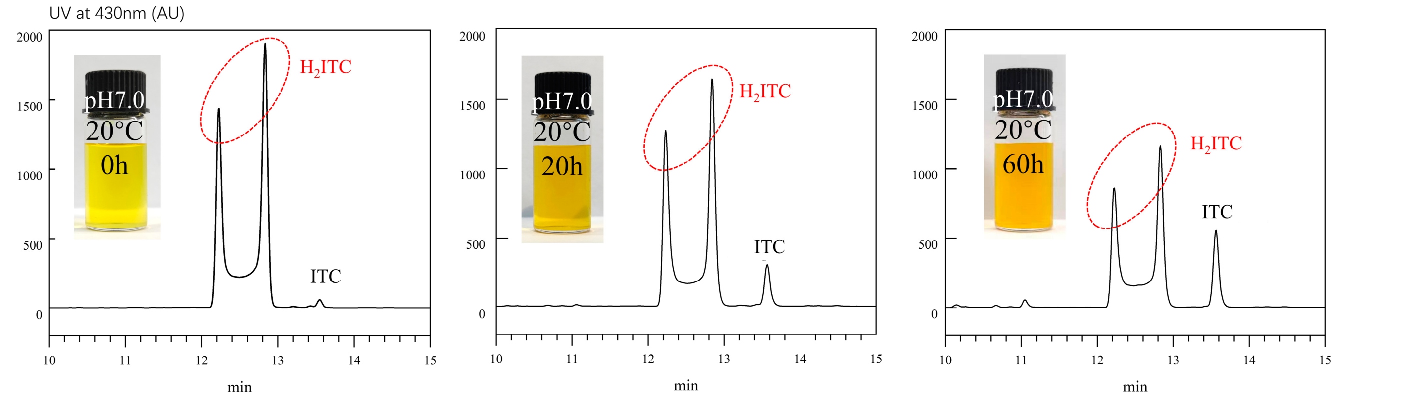

Figure S25. HPLC of 7,12-dihydroisatropolone C (in 30% MeOH) changing to isatropolone C at pH7.0 plus 20°C.

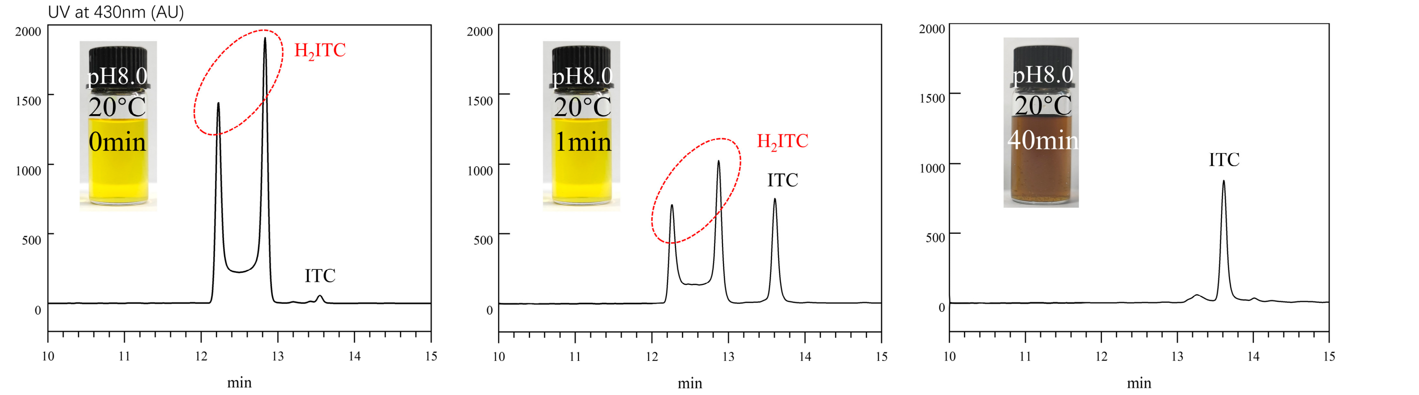

Figure S26. HPLC of 7,12-dihydroisatropolone C (in 30% MeOH) changing to isatropolone C at pH8.0 plus 20°C.

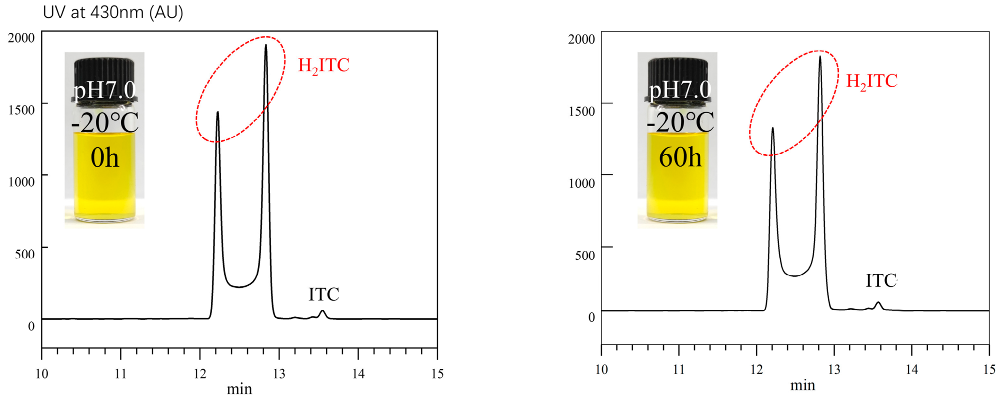

Figure S27. HPLC of 7,12-dihydroisatropolone C (in 30% MeOH) changing to isatropolone C at pH7.0 plus −20°C.

For analytical HPLC of Figures S25-S27, a reverse-phase C18 column (YMC-Pack ODS-A column: 250 mm × 4.6 mm, S-5 μm, 12 nm) was used with a gradient solvent system from 15% to 70% CH_3CN-H_2O (0.1% HAc, v/v), 1.0 mL/min. The injection volume is 30 μl for each sample.

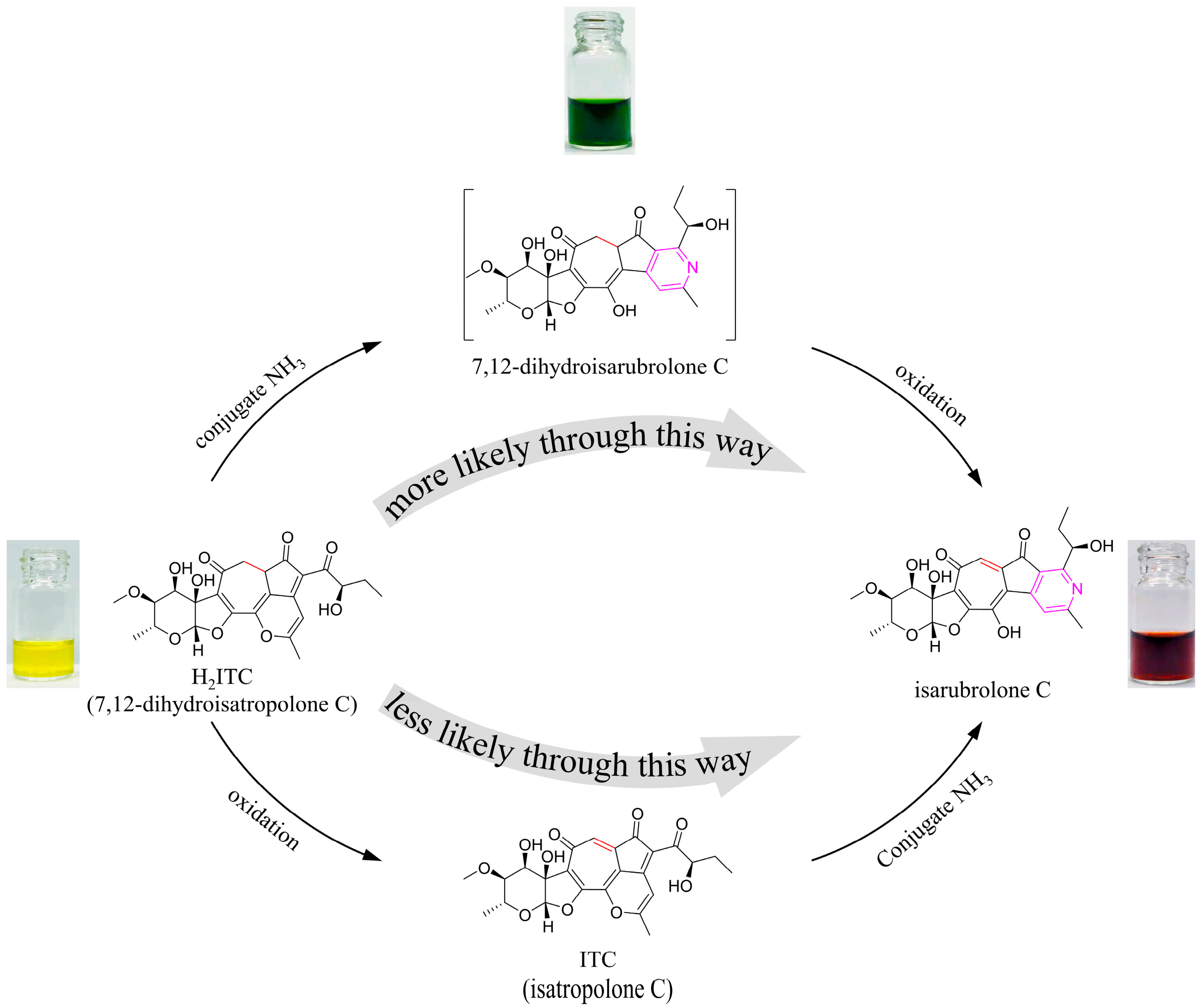

Figure S28. H_2ITC conjugates NH_3 for 7,12-dihydroisarubrolone C production.

A brief description about reaction: H_2ITC was dissolved in 1.0 ml 30% MeOH/H_2O (in a 2.0 ml vial) at concentration 0.45 mg/ml, and kept at room temperature (20°C). A volume of 0.5 ml ammonia water (13 mol/L) was dropped into the vial with shaking. The solution changed color from light-yellow to dark green quickly and then to purple-red slowly (within a few minutes). The expected 7,12-dihydroisarubrolone C was not observed by HPLC (and LC-MS) analysis of the dark green solution and the purple-red solution. Instead, isarubrolone C (purple-red) was only observed. The reaction was also repeated under argon protection with a similar result.

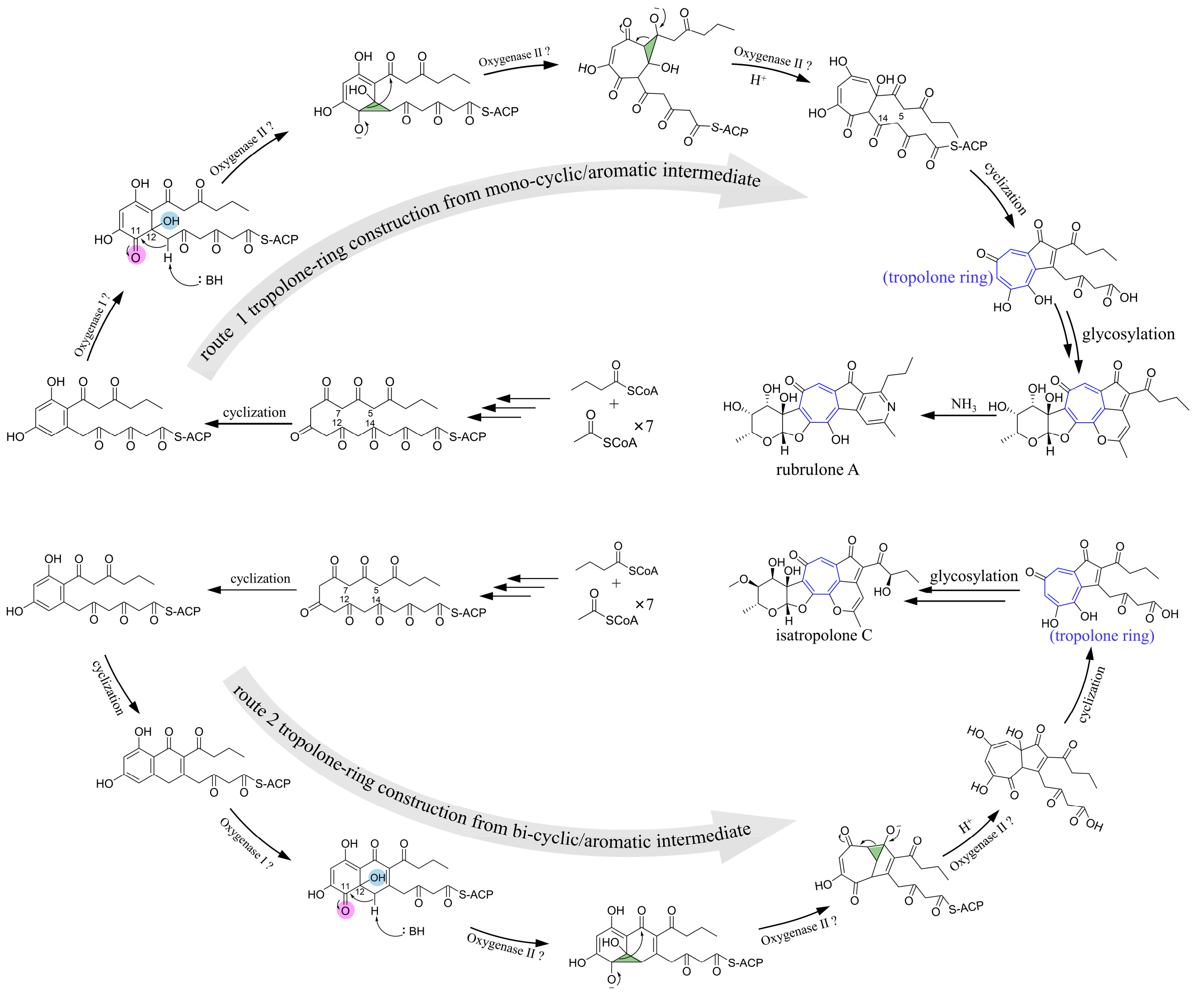

Scheme S1. Biosynthesis of rubrulone A focusing on oxidative rearrangement for tropolone-ring construction from mono-cyclic/aromatic intermediate proposed by Yan *et al* (route 1), and biosynthesis of isatropolone C (ITC) focusing on oxidative rearrangement for tropolone-ring construction from bi-cyclic/aromatic intermediate proposed by Cai *et al* (route 2).

Yan, Y.; Ma, Y. T.; Yang, J.; Horsman, G. P.; Luo, D.; Ji, X.; Huang, S. X., Tropolone Ring Construction in the Biosynthesis of Rubrolone B, a Cationic Tropolone Alkaloid from Endophytic *Streptomyces*. *Org Lett* **2016**, 18, 1254-7.

Cai, X.; Shi, Y. M.; Pohlmann, N.; Revermann, O.; Bahner, I.; Pidot, S. J.; Wesche, F.; Lackner, H.; Buchel, C.; Kaiser, M.; Richter, C.; Schwalbe, H.; Stinear, T. P.; Zeeck, A.; Bode, H. B., Structure and Biosynthesis of Isatropolones, Bioactive Amine-Scavenging Fluorescent Natural Products from *Streptomyces* Gö66. *Angew Chem Int Ed Engl* **2017**, 56, 4945-4949.

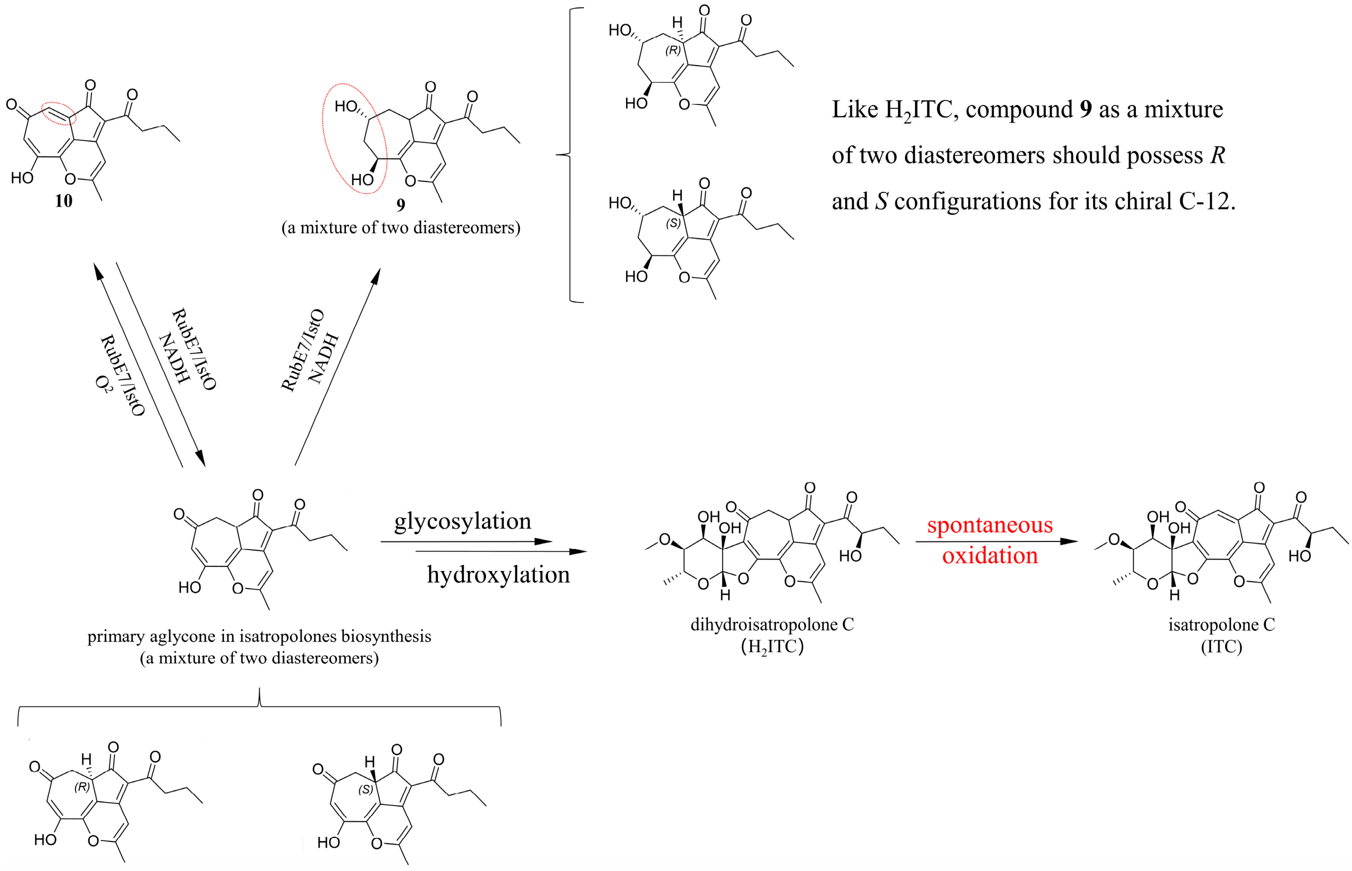

Figure S29. Two compounds **9-10** characterized from *S. lividans* heterologous-expressing *istG-R* for the aglycone biosynthesis of isatropolone (reported by Cai *et al.*)

Due to our discovery of H_2ITC, we speculate the primary aglycone in isatropolones biosynthesis may be the dihydrogenated **10**

Cai, X.; Shi, Y. M.; Pohlmann, N.; Revermann, O.; Bahner, I.; Pidot, S. J.; Wesche, F.; Lackner, H.; Buchel, C.; Kaiser, M.; Richter, C.; Schwalbe, H.; Stinear, T. P.; Zeeck, A.; Bode, H. B., Structure and Biosynthesis of Isatropolones, Bioactive Amine-Scavenging Fluorescent Natural Products from *Streptomyces* Gö66. *Angew Chem Int Ed Engl* **2017**, 56, 4945-4949.
